# Evidence from a meta-analysis and systematic review reveals the global prevalence of mild cognitive impairment

**DOI:** 10.3389/fnagi.2023.1227112

**Published:** 2023-10-27

**Authors:** Wen-xin Song, Wei-wei Wu, Yuan-yuan Zhao, Hai-lun Xu, Guan-cheng Chen, Shan-yu Jin, Jie Chen, Shao-xiang Xian, Jing-hong Liang

**Affiliations:** ^1^The First Clinical Medical College of Guangzhou University of Chinese Medicine, Guangzhou, China; ^2^Gannan Medical University, Ganzhou, China; ^3^The First Affiliated Hospital of Guangzhou University of Chinese Medicine, Guangzhou, China; ^4^Department of Maternal and Child Health, School of Public Health, Sun Yat-sen University, Guangzhou, China

**Keywords:** mild cognitive impairment, global prevalence, COVID-19, meta-analysis, systematic review

## Abstract

**Objective:**

Mild cognitive impairment (MCI) is a preclinical and transitional stage between healthy ageing and dementia. The purpose of our study was to investigate the recent pooled global prevalence of MCI.

**Methods:**

This meta-analysis was in line with the recommendations of Cochrane’s Handbook and Preferred Reporting Items for Systematic Reviews and Meta-Analyses (PRISMA) 2020. We conducted a comprehensive search using the PubMed, Embase, Web of Science, CNKI, WFD, VIP, and CBM from their inception to March 1, 2023. Quality assessment was guided by the Agency for Healthcare Research and Quality (AHRQ) methodology checklist. The pooled global prevalence of MCI was synthesized using meta-analysis via random effect model. Subgroup analyses were performed to examine considered factors potentially associated with MCI prevalence.

**Results:**

We identified 233 studies involving 676,974 individuals aged above 50 years. All the studies rated as moderated-to-high quality. The overall prevalence of MCI was 19.7% [95% confidence interval (95% CI): 18.3–21.1%]. Subgroup analyses revealed that the global prevalence of MCI increased over time, with a significant rise [32.1% (95% CI: 22.6–41.6%)] after 2019. Additionally, MCI prevalence in hospitals [34.0% (95% CI: 22.2–45.7%)] was higher than in nursing homes [22.6% (95% CI: 15.5–29.8%)] and communities [17.9% (95% CI: 16.6–19.2%)], particularly after the epidemic of coronavirus disease 2019 (COVID-19).

**Conclusion:**

The global prevalence of MCI was 19.7% and mainly correlated with beginning year of survey and sample source. The MCI prevalence increased largely in hospitals after 2019 may be related to the outbreak of COVID-19. Further attention to MCI is necessary in the future to inform allocation of health resources for at-risk populations.

## 1. Introduction

Mild cognitive impairment (MCI) is a condition characterized by mild cognitive deficit, while still retains the ability to perform daily living activities (Petersen et al., 2014). A recent review reported that up to 15.56% of community dwellers aged over 50 years were affected by MCI worldwide (Bai et al., 2022). MCI is considered as a symptomatic precursor of dementia, serving as an intermediate stage between normal aging and dementia. Over 46% of individuals with MCI progressed to clinical dementia within 3 years, which is one of the major causes of disability and dependency among older people (Trambaiolli et al., 2021). Therefore, MCI as predementia imposes potential economic burden on individuals, families, and society (Wang et al., 2022).

MCI is currently viewed as an “intervention window” for delaying the onset of dementia (Anderson, 2019; Liang et al., 2019; Wang et al., 2020). Understanding the global prevalence of MCI is essential for developing relevant strategies to prevent dementia. In recent years, several epidemiological studies have been conducted on MCI prevalence at different levels. For instance, Bai et al. revealed that the prevalence of MCI among community dwellers worldwide was over 15% and influenced by factors such as age, sex, educational level, and sample source (Bai et al., 2022). Deng et al. reported a prevalence rate of MCI in China was 15.4%, which was associated with unhealthy lifestyles such as alcohol consumption and lack of exercise, as well as health conditions like diabetes, hypertension, coronary heart disease, and depression (Deng et al., 2021). This information is crucial for developing prevention strategies aimed at addressing these risk factors. However, there are significant heterogeneities among previous studies. First, some studies may reveal the partial results when investigating the prevalence of MCI among the global population. On the one hand, differences in population characteristics could lead to variation in prevalence. Specifically, populations with the high-risk diseases, such as diabetes and depression, have a higher MCI prevalence (Hasche et al., 2010; Bo et al., 2015), which could affect the accuracy of total prevalence in healthy individuals. On the other hand, differences in geographical distribution could also affect the precision of global MCI prevalence when investigators omitted evidence from other geographical areas and sample source (Bai et al., 2022; Chen et al., 2023). Second, during the same period and in the same region, different studies have reported significant disparities in results. For instance, two studies from China in 2019 produced significantly different prevalence: one reported 9.67% (Ruan et al., 2020), while the other reported 27.8% (Lu et al., 2019). Similarly, two studies conducted 1 year apart reported nearly a threefold difference in MCI prevalence results in China: one reported 33.3% in 2015, while the other reported 10.42% in 2016 (McGrattan et al., 2021). These discrepancies may be attributed to variations in study design, such as search sources, screening tools, and diagnostic criteria for MCI. Lastly, the outbreak of the coronavirus disease 2019 (COVID-19) has significantly impacted society, affecting the lifestyle and health of everyone. There is evidence suggesting that some patients who have recovered from COVID-19 exhibit cognitive deficits (Liu et al., 2021; Crivelli et al., 2022). Consequently, the prevalence of neurological diseases, including MCI, may be even more severe as a result of COVID-19. However, whether COVID-19 has increased MCI prevalence remains unknown, highlighting the need for more updated research into the prevalence of MCI. Therefore, a comprehensive and updated meta-analysis on the global prevalence of MCI is urgently needed to identify the risk factors and provide a reference for researchers and clinicians. The purpose of this study is to investigate the recent global prevalence of MCI among the widest possible population.

## 2. Methods

This systematic review was conducted in accordance with the recommendations of Cochrane’s Handbook (Cumpston et al., 2019) and the Systematic Reviews and Meta-Analyses (PRISMA) 2020 (Page et al., 2021) (Supplementary File S2). These analyses relied solely on previously published studies, so ethical approval or patient consent was not required.

### 2.1. Search strategies

The eligible studies were identified through a comprehensive literature search in PubMed, Embase, Web of Science, CNKI, WFD, VIP, and CBM databases from their inception to March 1, 2023. A search strategy was employed using Medical Subject Headings (MeSH) terms associated with keywords and Boolean operators on “cognitive dysfunction,” “mild cognitive impairment,” “mild cognitive disorder,” “prevalence,” “epidemiology,” and “epidemiological study” et al. In addition, manual retrieval was performed on the reference lists of relevant reviews and meta-analysis to search for additional studies on MCI prevalence. All database specific search queries could be found in Supplementary File S1.

### 2.2. Inclusion and exclusion criteria

Inclusion criteria were developed based on the PICOS principle, including participants (P), outcomes (O), and study design (S):

1. Participants: Studies were included when participants were diagnosed with MCI using recognized criteria, such as Petersen criteria (P-MCI) (Ronald, 2011), Diagnostic and Statistical Manual of Mental Disorders (DSM) (Sharp, 2022), etc.
2. Outcomes: Prevalence of MCI (or any of MCI subtypes) or data regarding the prevalence of MCI were provided in the report. If multiple articles were published based on the same dataset, only the most recent study was included.
3. Study design: Our study included all types of cohort and cross-sectional studies without any restriction in language, region, or publication date.

Studies were excluded if they met the following conditions:

1. Participants: Studies involving other types of cognitive dysfunction, such as dementia.
2. Outcomes: Studies involving the prevalence of comorbidity with MCI, such as hypertension, coronary heart disease, and depression.
3. Study design: Randomized controlled trials (RCT), systematic reviews, meta-analysis, case–control studies, bibliographic review articles, letters to the editor, and articles published only in abstract form.
4. Full texts or data could not be obtained for our analyses.

### 2.3. Literature selection and data extraction

All citations were downloaded and managed using EndNote X9 software (Thompson ISI Research Soft, Clarivate Analytics, Philadelphia, United States). First, duplicate items were retrieved and removed. Then, based on inclusion and exclusion criteria, three investigators (WXS, YYZ, and HLX) independently reviewed the titles, abstracts, and full texts of publications to exclude irrelevant studies. All the eligible citations were cross-checked again to ensure accuracy. The relevant key data from the included studies were extracted into Microsoft Excel worksheets: (1) basic information: first author, publication year; (2) baseline characteristics: sample size, cases, age, proportion of males, beginning of survey, diagnostic criteria, region. The corresponding authors were consulted to obtain the essential information missing in the original studies.

### 2.4. Quality assessment

Three researchers (WXS, YYZ, and HLX) independently assessed the methodological quality of the included studies using the Agency for Healthcare Research and Quality (AHRQ) methodology checklist (Rostom et al., 2004). The checklist included 11 items: (I) Define the source of information; (II) List inclusion and exclusion criteria for exposed and unexposed subjects or provide a reference to previous publications that describe these criteria; (III) Indicate time period used for identifying patients; (IV) Indicate whether or not subjects were consecutive if not population-based; (V) Indicate if evaluators of subjective components of were masked to other aspects of the status of the participants; (VI) Describe any assessments undertaken for quality control purposes; (VII) Explain any patient exclusions from analysis; (VIII) Describe how confounding was assessed and/or controlled; (IX) If applicable, explain how missing data were handled in the analysis; (X) Summarize patient response rates and completeness of data collection; (XI) Clarify what follow-up, if any, was expected and the percentage of patients for which incomplete data or follow-up was obtained. The quality score for individual study ranges from 0 to 11, with 1 point for each item, and the study quality is separated into three levels: low (0–3), moderate (4–7), and high (8–11) (Hu et al., 2015). Any disagreements and uncertainty were resolved by discussion.

### 2.5. Statistical analyses

The overall prevalence and 95% confidence intervals (95% CI) was estimated using a random-effects model (Hedges, 1984). Heterogeneity was assessed by utilizing *I*^2^ statistics, with *I*^2^ > 50% or *p* < 0.1 indicating high heterogeneity (Higgins et al., 2003). A series of subgroup analyses were conducted to examine considered factors potentially associated with MCI prevalence. The subgroup variables included study type (cohort, cross-sectional), diagnostic method (P-MCI, DSM), male-to-female ratio (male/female ≥1, male/female <1), region^1^ (developing country, developed country), regions^2^ (Asia, Europe, North America, Africa, Oceania, South America), beginning year of survey (≤ 2009, 2010–2018, ≥ 2019), sample size (0–1,000, 1,001–5,000, 5,001–10,000, ≥10,001), sample source (community, nursing home, hospital), MCI subtype (aMCI/naMCI ≥1, aMCI/naMCI <1), basic diseases/non basic diseases (≥ 1, < 1) and the time trends in prevalence from different sample sources. Potential publication bias was assessed by using the funnel plot (Sedgwick, 2015) and Egger’s test (Egger et al., 2003). All the aforementioned sequences of analyses were performed in Stata version 15.0 (Nyaga et al., 2014) using “metan” and “metabias” packages.

## 3. Results

### 3.1. Literature selection

**Figure 1:**
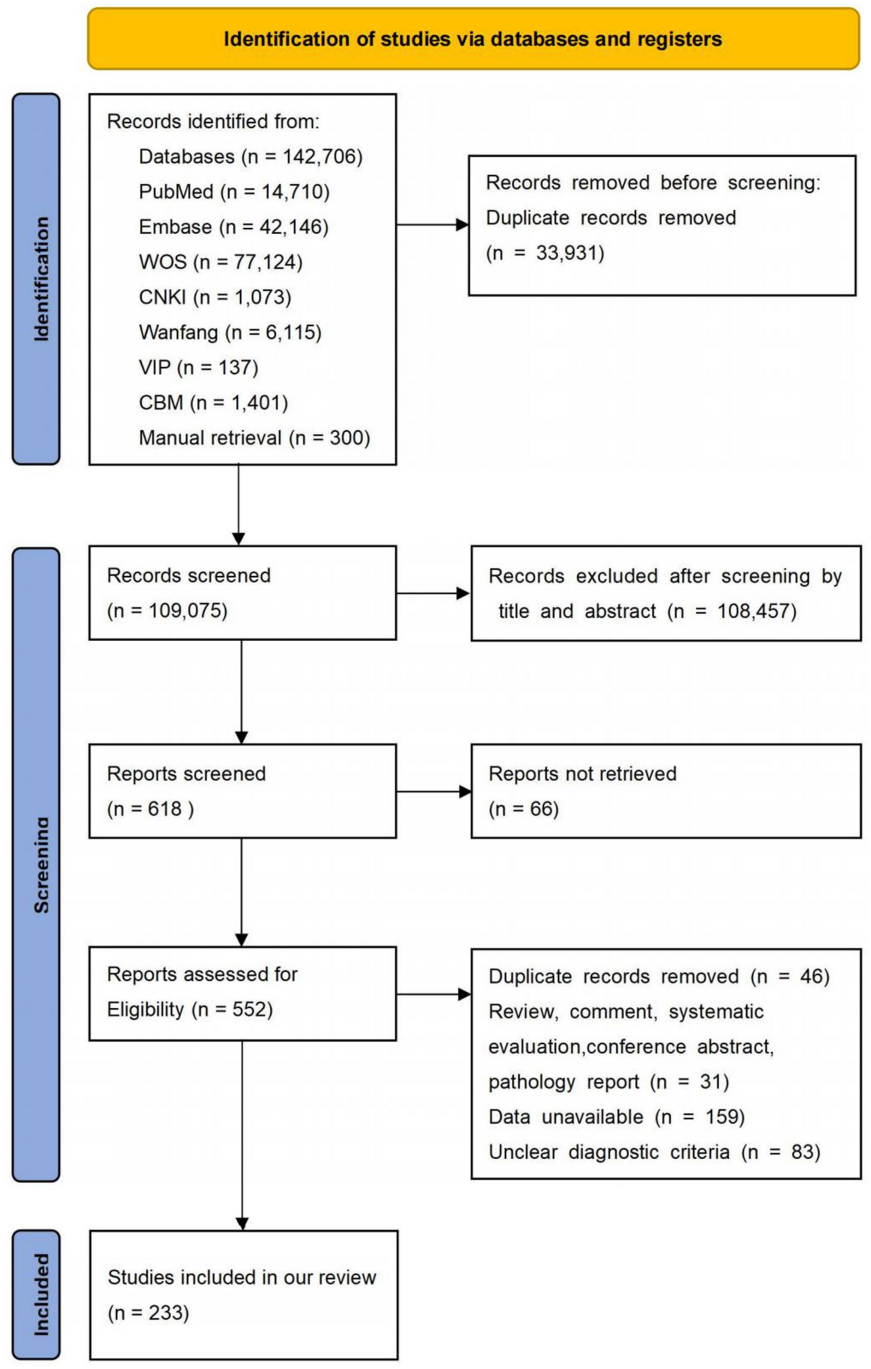
The screening process of the literature.

We initially obtained 143,006 studies, including 142,706 citations from databases and 300 additional studies from manual retrieval. Then, 33,931 studies were excluded for duplication, 108,457 articles were removed due to irrelevant titles and abstracts. Subsequently, 385 studies were excluded for various reasons: 66 were not available in full, 31 were non-observational studies (RCT, reviews, commentaries, systematic reviews, meta-analysis, conference abstracts, case reports), 159 had no available data, 83 had unclear diagnostic criteria, and 46 were reduplicated. Finally, 233 studies were included in this meta-analysis. The study selection process is shown in Figure 1. And all included studies in this systematic review and meta-analysis showed in Supplementary File S4.

### 3.2. Characteristics and quality of included studies

The 233 included studies were conducted between 1981 and 2021, enrolling 676,974 individuals aged from 50 to 107 years old. Most studies were cross-sectional studies (*N* = 207, 88.8%) and conducted in Asia (*N* = 171, 75.0%). The common diagnostic criteria for MCI was P-MCI (*N* = 150, 77.7%). Other detailed information on study characteristics is presented in Table 1.

**Table 1:** Characteristics of studies included in this meta-analysis.

ID	Study	Study design	Cases	Sample	Age, mean ± sd (range)	Proportion of males (%)	Beginning of survey	Diagnostic criteria	Region	Quality score
1	Björk et al. (2018)	Cross-sectional	1,067	4,545	85.50 ± 7.80	36.41%	2013–2014	P-MCI	Swedish	9
2	Tiwari et al. (2013)	Cross-sectional	98	2,146	≥60	47.44%	2008–2010	P-MCI	India	8
3	Rao et al. (2018)	Cross-sectional	299	2,111	≥65	40.50%	2009	P-MCI	China	8
4	VancamPfort et al. (2017)	Cross-sectional	5,005	32,715	≥50	48.30%	2007–2010	DSM-IV	China, Ghana, India, Mexico, Russia, South africa	7
5	Lu et al. (2019)	Cross-sectional	1,541	5,542	≥60	46.26%	2010 and 2015	P-MCI	China	7
6	Su et al. (2013)	Cross-sectional	145	796	≥60	32.79%	2012	P-MCI	China	4
7	Zhang et al. (2013)	Cross-sectional	450	2,460	60–89	45.98%	NR	P-MCI	China	5
8	Zhang et al. (2015)	Cross-sectional	651	1,971	≥60	37.44%	NR	DSM-IV	China	6
9	Li et al. (2013)	Cross-sectional	332	3,484	≥65	41.30%	2007–2009	P-MCI	China	9
10	Guo et al. (2013)	Cross-sectional	136	940	≥60	43.19%	NR	P-MCI	China	6
11	Yin et al. (2012)	Cross-sectional	67	1,011	≥65	40.55%	2007–2009	P-MCI	China	7
12	Pan et al. (2012)	Cross-sectional	154	897	69.68 ± 7.06	48.38%	2011–2011	P-MCI	China	9
13	Xia et al. (2006)	Cross-sectional	16	145	67.96 ± 6.49	39.31%	2000–2004	DSM-IV	China	7
14	Yang et al. (2017)	Cross-sectional	296	1,000	71.45 ± 5.86	48.60%	NR	P-MCI	China	7
15	Zhang et al. (2018)	Cohort study	430	1,033	≥55	33.69%	2016–2017	P-MCI	China	7
16	Jiang et al. (2019)	Cross-sectional	833	2,886	69.98 ± 5.90	41.61%	2017	P-MCI	China	7
17	Dai et al. (2019)	Cross-sectional	201	1,184	67.96 ± 6.49	50.17%	2019	CDGM	China	8
18	Liu et al. (2019)	Cross-sectional	73	554	≥60	64.80%	2018	CDGM	China	9
19	Yuan et al. (2019)	Cross-sectional	199	1,032	66 ± 7	38.19%	2015	P-MCI	China	6
20	Yuan et al. (2021)	Cross-sectional	613	3,043	≥60	51.36%	2016	P-MCI	China	8
21	Luo et al. (2015)	Cross-sectional	554	3,063	70.00 ± 7.70	45.60%	2010	P-MCI	China	6
22	Xu et al. (2014)	Cross-sectional	526	2,426	69.10 ± 6.80	39.30%	2010–2011	P-MCI	China	8
23	Tang et al. (2007)	Cross-sectional	217	1,865	60–100	48.10%	2004	P-MCI	China	6
24	Gang et al. (2008)	Cross-sectional	203	1,750	60–100	48.51%	2004	P-MCI	China	8
25	Huang et al. (2008)	Cross-sectional	257	4,697	≥60	41.15%	2001–2002	P-MCI	China	8
26	Ren et al. (2013)	Cross-sectional	99	946	≥60	50.74%	2011	DSM-IV	China	8
27	Zhou et al. (2011)	Cross-sectional	107	1,227	≥60	43.68%	2009–2010	DSM-IV	China	8
28	Chen et al. (2015)	Cross-sectional	352	1,695	≥60	46.90%	NR	P-MCI	China	4
29	Pan et al. (2012)	Cross-sectional	67	287	≥60	42.86%	NR	P-MCI	China	7
30	Song et al. (2012)	Cross-sectional	167	2,279	≥60	48.79%	2010–2011	P-MCI	China	8
31	Zhu et al. (2009)	Cross-sectional	148	1,511	≥60	45.40%	2008	DSM-IV	China	8
32	Wu et al. (2012)	Cross-sectional	396	1,583	≥60	50.28%	2011–2012	CDGM	China	7
33	Liao et al. (2012)	Cross-sectional	41	399	60–92	46.37%	NR	P-MCI	China	5
34	Zhang et al. (2014)	Cross-sectional	287	1,764	≥60	44.05%	2012	P-MCI	China	7
35	Afgin et al. (2012)	Cohort study	303	944	≥65	49.30%	NR	DSM-IV	Israel	10
36	Artero et al. (2008)	Cohort study	2,882	6,892	≥65	53.19%	1991–2001	DSM-IV	French	9
37	Lee et al. (2009)	Cohort study	188	927	≥60	33.66%	2005–2007	P-MCI	Korea	8
38	Ogunniyi et al. (2016)	Cross-sectional	111	613	72.90 ± 8.50	68.35%	2013–2014	DSM-IV and P-MCI	Nigerian	9
39	Petersen et al. (2010)	Cross-sectional	329	1,969	70–89	50.89%	2004–2007	DSM-IV	United States	11
40	Pilleron et al. (2015)	Cohort study	133	2,002	≥65	NR	2011–2012	P-MCI	Central Africa	6
41	Richard et al. (2013)	Cohort study	429	2,160	NR	NR	1999–2001	P-MCI	United States	8
42	Kumar et al. (2005)	Cohort study	93	2,518	NR	NR	2001–2002	P-MCI	Australia	9
43	Lee et al. (2009)	Cohort study	197	714	71.90 ± 5.70	42.16%	2005	P-MCI	Korea	6
44	Lee et al. (2012)	Cross-sectional	67	318	65.90 ± 5.30	40.88%	2008–2009	P-MCI	Malaysian	8
45	Purser et al. (2005)	Cohort study	810	3,673	74	38.69%	1981, 1984, 1987, and 1990	P-MCI	United States	8
46	De Jager et al. (2005)	Cohort study	40	157	NR	NR	NR	P-MCI	United Kingdom	7
47	Khedr et al. (2015)	Cross-sectional	12	691	≥60	NR	2011–2013	DSM-IV	Egypt	7
48	Yu et al. (2016)	Cohort study	66	376	68.60 ± 4.70	NR	NR	DSM-IV	China	5
49	Ma et al. (2016)	Cross-sectional	574	5,241	72.13 ± 4.22	43.90%	2012–2012	P-MCI	China	9
50	Wang et al. (2015)	Cross-sectional	625	3,136	69.30 ± 6.80	40.66%	2012–2012	P-MCI	China	8
51	Jia et al. (2013)	Cross-sectional	2,137	10,276	NR	42.41%	2008–2009	DSM-IV	China	9
52	Hu et al. (2012)	Cross-sectional	1,782	9,146	65.62 ± 7.52	43.83%	2008–2009	DSM-IV	China	7
53	Qiu et al. (2003)	Cross-sectional	92	3,910	66.97 ± 8.44	49.68%	2000–2001	P-MCI	China	8
54	Lei et al. (2008)	Cross-sectional	680	4,419	66.40 ± 5.60	41.68%	2005	The diagnostic criteria for MCI in Sweden, 2001	China	8
55	Lao et al. (2011)	Cross-sectional	326	7,665	≥55	45.78%	2010	P-MCI	China	5
56	Yang et al. (2011)	Cross-sectional	337	454	72.67 ± 6.34	69.16%	2009	Chinese guidelines and P-MCI	China	4
57	Yin et al. (2011)	Cross-sectional	310	2,164	≥60	45.84%	2010	P-MCI	China	6
58	Tong et al. (2013)	Cross-sectional	200	1,575	≥60	NR	2012	P-MCI	China	6
59	Xiong et al. (2013)	Cross-sectional	339	2,978	≥65	44.12%	2011	DSM-IV	China	7
60	Zhang et al. (2013)	Cross-sectional	450	2,460	≥60	45.98%	NR	P-MCI	China	8
61	Gu et al. (2014)	Cohort study	92	679	60–91	44.33%	2010–2013	IWG	China	7
62	Qin et al. (2014)	Cross-sectional	612	4,086	≥55	35.00%	2011–2012	P-MCI	China	8
63	Sun et al. (2016)	Cross-sectional	40	384	≥65	52.08%	NR	IWG and ADNI	China	4
64	Zhou et al. (2016)	Cross-sectional	221	804	60–88	46.52%	2014–2015	Chinese guidelines and P-MCI	China	7
65	Guo et al. (2012)	Cross-sectional	35	264	≥65	50.76%	2008–2009	P-MCI	China	8
66	Jia et al. (2014)	Cross-sectional	2,137	10,276	≥65	42.61%	2008–2009	DSM-IV	China	8
67	Li et al. (2013)	Cross-sectional	160	1,020	≥55	36.67%	NR	P-MCI	China	8
68	Ding et al. (2015)	Cross-sectional	601	2,985	≥60	NR	2010–2011	DSM-IV	China	8
69	Xu et al. (2014)	Cross-sectional	526	2,426	≥60	39.32%	2010–2011	P-MCI	China	8
70	Zanetti et al. (2006)	Cohort study	65	400	≥65	NR	2000	DSM-IV	Italy	7
71	Pioggiosi et al. (2006)	Cross-sectional	11	34	96.40 ± 3.90	20.59%	1994–1996	DSM-IV	Italy	7
72	Manly et al. (2005)	Cohort study	372	1,315	≥65	31.18%	NR	P-MCI	United States	6
73	Purser et al. (2005)	Cohort study	810	3,673	≥65	38.69%	1981–1991	P-MCI	United States	6
74	Kim et al. (2007)	Cohort study	388	1,215	≥60	42.80%	2004–2006	P-MCI	Korea	8
75	Jungwirth et al. (2005)	Cross-sectional	41	592	75	NR	2002	P-MCI	Australia	7
76	Das et al. (2007)	Cross-sectional	111	745	≥50	49.26%	2003–2004	DSM-IV	India	8
77	Tognoni et al. (2005)	Cross-sectional	79	1,600	≥65	40.38%	2000–2001	P-MCI	Italy	8
78	Boeve et al. (2003)	Cross-sectional	13	111	90–99	20.72%	1997–1999	P-MCI	United States	8
79	Ganguli et al. (2004)	Cohort study	40	1,248	NR	39.26%	1987–2001	P-MCI	United States	7
80	Ravaglia et al. (2008)	Cohort study	72	865	≥65	NR	1999–2004	IWG	United States	8
81	Xie et al. (2003)	Cross-sectional	54	311	≥75	100%	1998	P-MCI	NR	4
82	Yu et al. (2003)	Cross-sectional	216	2,674	≥60	60.96%	2001	DSM-IV	China	6
83	Wu et al. (2005)	Cross-sectional	45	267	≥80	37.08%	NR	Chinese guidelines and P-MCI	China	4
84	Yang et al. (2008)	Cross-sectional	647	3,175	≥60	38.33%	NR	Chinese guidelines and P-MCI	China	4
85	Liu et al. (2007)	Cross-sectional	838	2,944	≥60	84.65%	NR	Chinese guidelines and P-MCI	China	6
86	Wada-isoe et al. (2012)	Cohort study	211	723	77.80 ± 6.79	NR	2010	IWG	Japan	7
87	Vlachos et al. (2020)	Cohort study	243	1,960	≥65	40.61%	NR	P-MCI	Greece	4
88	Bickel et al. (2006)	Cross-sectional	287	794	65–85	40.68%	NR	DSM-IV	German	8
89	Busse et al. (2003)	Cohort study	116	1,045	NR	NR	1997–1998	P-MCI	German	6
90	Rahman et al. (2009)	Cross-sectional	104	268	60–76	54.48%	NR	DSM-IV	Egypt	5
91	Yu et al. (2003)	Cross-sectional	216	2,674	≥60	60.96%	NR	P-MCI	China	7
92	Assaf et al. (2021)	Cross-sectional	50	337	≥60	54.70%	NR	IWG	Lebanon	8
93	Eramudugolla et al. (2022)	Cohort study	132	1,427	60–64	44.11%	NR	DSM-IV	Australia	8
94	Hussenoeder et al. (2020)	Cross-sectional	110	903	86.50 ± 3.10	33.22%	2003–2013	IWG	Germany	8
95	Mooldijk et al. (2022)	Cohort study	648	7,058	≥60	42.87%	2002–2014	P-MCI	Netherland	8
96	Nakahata et al. (2021)	Cross-sectional	191	2,286	69	NR	2014–2017	NIA-AA	Japan	7
97	Samson et al. (2022)	Cross-sectional	255	506	55–93	47.23%	NR	P-MCI	United States	8
98	Lee et al. (2022)	Cross-sectional	2,520	13,623	≥65	45.50%	2007–2010	DSM-IV	China, Ghana, India, Mexico, Russia, South Africa	7
99	Smith et al. (2022)	Cross-sectional	5,005	32,715	50–65	48.30%	2007–2010	DSM-IV	China, Ghana, India, Mexico, Russia, South Africa	7
100	Xu et al. (2021)	Cross-sectional	55	171	70.68 ± 7.92	49.12%	2010–2010	P-MCI	China	7
101	Yamane et al. (2022)	Cross-sectional	61	865	≥65	38.96%	2014–2017	P-MCI	Japan	4
102	Yang et al. (2021)	Cross-sectional	276	925	71.16 ± 4.41	NR	NR	DSM-IV	China	7
103	Yu et al. (2022)	Cross-sectional	86	163	81.20 ± 4.70	28.83%	2018–2021	ADNI	Spanish	8
104	Tang al. 2007	Cross-sectional	217	1,865	≥60	48.10%	2004–2004	P-MCI	China	7
105	Gjøra et al. (2021)	Cross-sectional	3,382	9,663	≥70	43.25%	2017–2019	DSM-V	Swedish	9
106	Ramlall et al. (2013)	Cross-sectional	38	140	75.20 ± 8.90	30.71%	NR	IWG	South Africa	6
107	Yang et al. (2019)	Cross-sectional	318	2,015	79.5	NR	2014	NIA-AA	China	10
108	Amoo et al. (2020)	Cross-sectional	397	532	71.40 ± 8.86	35.30%	NR	P-MCI	Nigera	5
109	Bae et al. (2017)	Cross-sectional	698	3,312	NR	44.17%	NR	IWG	Japan	6
110	Fernández-Blázquez et al. (2021)	Cross-sectional	83	1,180	74.90 ± 3.90	36.44%	2011	NIA-AA	Spanish	8
111	Ganguli et al. (2010)	Cross-sectional	697	1,982	77.60 ± 7.40	38.90%	NR	P-MCI	United States	6
112	González et al. (2019)	Cross-sectional	5,851	59,714	63.00 ± 6.80	45.00%	NR	NIA-AA	Spanish	8
113	Guaita et al. (2015)	Cross-sectional	65	1,321	71.68 ± 1.43	54.05%	NR	P-MCI	Italy	8
114	Heywood et al. (2017)	Cross-sectional	507	2,599	≥55	36.24%	2006–2009	P-MCI	Singapore	9
115	Kivipelto et al. (2001)	Cross-sectional	82	1,352	65–79	37.87%	NR	P-MCI	Finland	6
116	Lara et al. (2016)	Cross-sectional	348	3,625	66.26 ± 0.18	45.32%	NR	NIA-AA	Spanish	6
117	Chong et al. (2019)	Cross-sectional	158	1,209	68.08 ± 5.63	49.96%	NR	P-MCI	Malaysia	6
118	Das et al. (2007)	Cross-sectional	111	745	66.75 ± 9.96	49.26%	2003–2004	P-MCI	India	7
119	Juarez- Cedillo et al. (2012)	Cross-sectional	190	2,944	71.00 ± 7.10	42.19%	NR	P-MCI	Mexico	7
120	Ding et al. (2015)	Cross-sectional	601	3,141	73.30 ± 8.60	45.78%	NR	P-MCI	China	9
121	Jia et al. (2014)	Cross-sectional	2,137	13,806	≥65	31.72%	NR	P-MCI	China	8
122	Jia et al. (2020)	Cross-sectional	7,215	46,011	70.00 ± 7.51	49.70%	2015–2018	NIA-AA	China	11
123	Anstey et al. (2013)	Cross-sectional	141	2,551	68–72	39.98%	1999–2007	P-MCI	Australia	8
124	Dimitrov et al. (2012)	Cross-sectional	37	605	73.20 ± 5.70	42.98%	NR	P-MCI	Bulgaria	6
125	Gavrila et al. (2009)	Cross-sectional	88	1,074	74.30 ± 6.50	48.23%	2003–2005	P-MCI	Spanish	6
126	Han et al. (2017)	Cross-sectional	305	755	≥65	NR	2012	P-MCI	Korea	7
127	Hänninen et al. (2002)	Cross-sectional	43	806	68.10 ± 4.50	39.83%	NR	P-MCI	Finland	6
128	Juncos-Rabadán et al. (2012)	Cross-sectional	169	580	≥50	30.86%	NR	P-MCI	Spanish	5
129	Kim et al. (2011)	Cross-sectional	1,455	6,141	≥65	39.81%	2008	P-MCI	Korea	5
130	Limongi et al. (2017)	Cross-sectional	505	2,337	74	41.68%	2002–2004	P-MCI	Italy	9
131	Liu et al. (2022)	Cross-sectional	122	1,010	≥60	31.49%	2011–2016	P-MCI	Singapore	8
132	Lopez-Anton et al. (2015)	Cross-sectional	323	4,803	≥65	NR	NR	DSM-IV	Spanish	6
133	Luck et al. (2007)	Cross-sectional	499	3,242	≥75	34.42%	2003–2004	IWG	Germany	9
134	Mohan et al. (2019)	Cross-sectional	111	426	69.90 ± 7.90	38.03%	2012–2014	P-MCI	India	8
135	Mooi et al. (2016)	Cross-sectional	1,442	2,112	68.80 ± 6.10	48.58%	2013–2014	P-MCI	Malaysia	8
136	Moretti et al. (2013)	Cross-sectional	3,351	7,930	61–107	39.66%	NR	IWG and P-MCI	Italy	9
137	Noguchi-Shinohara et al. (2013)	Cross-sectional	107	650	76	40.46%	NR	IWG and P-MCI	Japan	7
138	Peltz et al. (2012)	Cross-sectional	70	420	≥90	34.05%	2003 and 2008	DSM-IV	USA	5
139	Robertson et al. (2019)	Cross-sectional	964	1,721	≥65	40.44%	2008–2011	DSM-IV	Canada	6
140	Sasaki et al. (2009)	Cross-sectional	557	1,433	≥65	NR	2001–2002	DSM-IV	Japan	5
141	Shahnawaz et al. (2013)	Cross-sectional	299	767	70–90	43.55%	NR	IWG	Australia	4
142	Teh et al. (2021)	Cross-sectional	32	2,165	≥60	45.87%	2012–2013	IWG and P-MCI	Singapore	7
143	Tsoy et al. (2019)	Cross-sectional	201	662	≥60	24.32%	NR	IWG	Kazakhstan	8
144	Vlachos et al. (2020)	Cross-sectional	243	1,960	73.46 ± 5.47	40.61%	NR	IWG and P-MCI	Greece	6
145	Liu et al. (2022)	Cross-sectional	5,432	10,432	≥65	47.68%	2011–2013	ADNI	China	7
146	Su et al. (2014)	Cross-sectional	145	796	≥60	32.79%	NR	P-MCI	China	6
147	Mías et al. (2007)	Cross-sectional	102	418	≥50	22.01%	2004–2005	P-MCI	Argentina	8
148	Pedraza et al. (2017)	Cross-sectional	421	1,235	≥50	24.78%	NR	P-MCI	Bogotá	8
149	Sánchez et al. (2019)	Cross-sectional	63	352	≥60	27.05%	NR	P-MCI	Peru	7
150	Monteagudo Torres et al. (2009)	Cross-sectional	19	201	≥60	NR	2006–2007	P-MCI	Cuba	6
151	Wesseling et al. (2013)	Cross-sectional	35	401	≥65	39.65%	2010–2011	P-MCI	Costa Rica	7
152	Li et al. (2020)	Cohort study	535	3,135	71.58 ± 8.06	NR	2011–2012	P-MCI	China	9
153	Rao et al. (2018)	Cross-sectional	299	2,111	≥65	40.50%	NR	P-MCI	China	7
154	Sun et al. (2014)	Cross-sectional	1,957	10,432	≥65	47.70%	NR	ADNI	China	5
155	Xiao et al. (2016)	Cohort study	267	1,068	72.80 ± 8.50	42.23%	NR	P-MCI	China	9
156	Liu et al. (2018)	Cross-sectional	317	1,796	≥60	46.05%	NR	DSM-IV	China	6
157	Wu et al. (2017)	Cross-sectional	371	1,846	69.52 ± 6.86	46.64%	2013–2014	P-MCI	China	8
158	Chuang et al. (2021)	Cross-sectional	82	470	71.20 ± 5.40	38.72%	2017–2019	NIA-AA	China	7
159	Janelidze et al. (2018)	Cross-sectional	113	851	56.50 ± 11.80	37.02%	NR	DSM-IV	Georgia	6
160	Pilleron et al. (2015)	Cross-sectional	266	2,002	≥65	NR	2011–2012	P-MCI and DSM-IV	South Africa	8
161	Vancampfort et al. (2017)	Cross-sectional	5,005	32,715	62.10 ± 15.60	48.30%	NR	P-MCI	China, Ghana, India, Mexico, Russia, South africa	9
162	Koyanagi et al. (2019)	Cross-sectional	312	3,672	≥50	44.01%	2007–2008	P-MCI	South Africa	7
163	Li et al. (2013)	Cross-sectional	160	1,020	63.90 ± 6.60	36.67%	NR	P-MCI	China	8
164	Kang et al. (2016)	Cross-sectional	180	1,248	≥60	51.68%	2015–2016	P-MCI	China	6
165	Huang et al. (2021)	Cross-sectional	1,830	5,103	≥55	44.95%	2018–2019	P-MCI	China	6
166	Bai et al. (2021)	Cross-sectional	92	428	86.34 ± 3.57	28.97%	2018–2019	P-MCI	China	6
167	Lu et al. (2022)	Cross-sectional	47	260	≥60	53.46%	2021	CGDM	China	6
168	Shi et al. (2019)	Cross-sectional	175	513	40–98	86.74%	2015–2019	P-MCI	China	6
169	Liu et al. (2005)	Cross-sectional	88	410	≥60	35.12%	2004	P-MCI	China	5
170	Sun et al. (2013)	Cross-sectional	53	471	83.00 ± 3.50	97.45%	2009–2010	IWG and P-MCI	China	7
171	Hai et al. (2010)	Cross-sectional	61	202	82.51 ± 2.14	74.26%	2007	IWG and P-MCI	China	6
172	Yuan et al. (2017)	Cross-sectional	158	1,013	60–96	52.82%	2014–2016	P-MCI.	China	8
173	Ji et al. (2017)	Cross-sectional	318	3,200	≥60	49.76%	NR	P-MCI	China	4
174	Wang et al. (2013)	Cross-sectional	199	1,033	≥55	38.14%	NR	P-MCI	China	6
175	Zhao et al. (2015)	Cross-sectional	171	976	≥60	46.82%	2013–2014	P-MCI	China	5
176	Li et al. (2013)	Cross-sectional	115	1,226	≥60	46.74%	NR	P-MCI	China	5
177	Pan et al. (2020)	Cross-sectional	214	1,012	≥60	47.23%	2015	P-MCI	China	6
178	Yu et al. (2012)	Cross-sectional	168	1,086	84.80 ± 4.40	100%	2010	IWG	China	7
179	Yu et al. (2002)	Cross-sectional	123	1,630	65–92	100%	2001	P-MCI	China	6
180	Cai et al. (2010)	Cross-sectional	105	1,498	≥60	NR	2004–2005	P-MCI	China	7
181	Chen et al. (2009)	Cross-sectional	195	925	≥60	40.65%	NR	P-MCI	China	5
182	Zhang et al. (2013)	Cross-sectional	86	321	81.55 ± 4.14	100%	2009	P-MCI	China	6
183	Sun et al. (2008)	Cross-sectional	45	536	72.60 ± 5.60	79.85%	2005	P-MCI and DSM-IV	China	5
184	Yu et al. (2004)	Cross-sectional	36	420	73.60 ± 5.60	74.29%	NR	P-MCI and DSM-IV	China	4
185	Zhang et al. (2008)	Cross-sectional	104	586	75.92 ± 4.35	70.48%	2005–2007	P-MCI and DSM-IV	China	6
186	Jiang et al. (2019)	Cross-sectional	833	2,886	≥60	41.61%	2017–2017	P-MCI and DSM-IV	China	8
187	Hu et al. (2012)	Cross-sectional	1,782	9,146	≥55	43.83%	2008–2009	DSM-IV	China	6
188	Guo et al. (2013)	Cross-sectional	178	1,367	≥60	49.60%	2011	DSM-IV	China	5
189	Li et al. (2015)	Cross-sectional	260	1,971	≥60	37.39%	NR	DSM-IV	China	5
190	Fan et al. (2014)	Cross-sectional	73	213	65.70 ± 6.08	36.15%	2012	P-MCI and DSM-IV	China	5
191	Lv et al. (2016)	Cross-sectional	95	820	60–85	47.68%	NR	P-MCI and DSM-IV	China	6
192	Zhang et al. (2021)	Cross-sectional	253	309	58.85 ± 0.58	53.40%	2019	P-MCI	China	7
193	Yuan et al. (2013)	Cross-sectional	631	3,311	≥60	32.47%	NR	P-MCI	China	6
194	Fang et al. (2015)	Cross-sectional	137	1,059	≥60	46.18%	NR	P-MCI	China	5
195	Pan et al. (2021)	Cross-sectional	326	734	≥60	40.74%	2019	P-MCI	China	5
196	Tao et al. (2016)	Cross-sectional	1,546	9,121	70.50 ± 7.68	53.95%	2013–2014	P-MCI	China	7
197	Li et al. (2021)	Cross-sectional	177	413	≥60	41.65%	2019	P-MCI	China	5
198	Xu et al. (2001)	Cross-sectional	417	1,516	≥65	NR	NR	P-MCI	China	5
199	Zhou et al. (2020)	Cross-sectional	49	114	81.30 ± 7.87	55.26%	2018–2019	P-MCI	China	4
200	Qiu et al. (2018)	Cross-sectional	65	239	65.68 ± 6.16	49.79%	NR	P-MCI	China	4
201	Xia et al. (2011)	Cross-sectional	47	20,367	NR	NR	2009–2019	DSM-IV	China	4
202	Wang et al. (2015)	Cross-sectional	236	718	NR	47.63%	2013–2014	ADNI	China	4
203	Zhang et al. (2020)	Cross-sectional	260	1,614	≥60	60.22%	2019–2019	P-MCI	China	4
204	Gao et al. (2011)	Cross-sectional	243	1,773	≥60	44.21%	2010–2011	P-MCI	China	8
205	Xue et al. (2010)	Cross-sectional	93	1,713	≥60	NR	2006	P-MCI	China	6
206	Zhou et al. (2010)	Cross-sectional	136	1,065	≥60	43.29%	NR	DSM-IV	China	5
207	Liang et al. (2008)	Cross-sectional	220	2,895	≥60	50.09%	NR	P-MCI	China	4
208	He et al. (2013)	Cross-sectional	69	598	60–90	71.57%	2011–2012	P-MCI	China	5
209	Zhang et al. (2014)	Cross-sectional	152	826	67.50 ± 7.03	60.65%	2012	P-MCI	China	5
210	Sun et al. (2012)	Cross-sectional	131	505	75.91 ± 7.96	34.46%	2011–2012	P-MCI	China	5
211	Sun et al. (2019)	Cross-sectional	402	2,105	74.35 ± 6.92	67.70%	2018	P-MCI	China	6
212	Xiong et al. (2013)	Cross-sectional	339	2,978	≥65	44.12%	NR	Chinese guidelines and P-MCI	China	4
213	Zhao et al. (2015)	Cross-sectional	174	1,598	≥60	54.26%	NR	DSM-IV	China	5
214	Sun et al. (2013)	Cross-sectional	74	427	79.17 ± 7.22	38.64%	2011	P-MCI	China	5
215	Song et al. (2019)	Cross-sectional	85	106	64.99 ± 7.05	NR	1987–2017	Chinese guidelines and P-MCI	China	6
216	Wu et al. (2017)	Cross-sectional	371	1,996	69.50 ± 6.86	46.39%	NR	P-MCI	China	7
217	Yang et al. (2016)	Cross-sectional	340	1,218	≥65	44.01%	NR	Chinese guidelines and P-MCI	China	5
218	Su et al. (2016)	Cross-sectional	145	796	≥60	32.79%	NR	P-MCI	China	5
219	Xiang et al. (2009)	Cross-sectional	72	532	≥60	47.37%	NR	Chinese guidelines and P-MCI	China	5
220	Xu et al. (2010)	Cross-sectional	571	2,161	≥60	50.49%	2007–2009	Chinese guidelines and P-MCI	China	6
221	Ma et al. (2019)	Cross-sectional	224	1,005	≥60	41.69%	2017–2018	P-MCI	China	5
222	An et al. (2020)	Cross-sectional	396	3,247	71.58 ± 5.41	45.64%	2019	Chinese guidelines and P-MCI	China	6
223	Yang et al. (2019)	Cross-sectional	319	2,015	≥65	NR	2014	NIA-AA	China	7
224	Liu et al. (2022)	Cross-sectional	69	476	≥60	45.38%	2018–2021	CDGM	China	7
225	Wang et al. (2017)	Cross-sectional	209	1,781	≥60	39.53%	2015	P-MCI	China	6
226	Wang et al. (2017)	Cross-sectional	25	84	≥60	60.71%	2015	P-MCI	China	6
227	Liu et al. (2021)	Cross-sectional	64	287	≥65	50.17%	2019–2020	Chinese guidelines and P-MCI	China	6
228	Zhou et al. (2013)	Cross-sectional	59	218	≥60	49.08%	2012	Chinese guidelines and P-MCI	China	5
229	Jia et al. (2020)	Cross-sectional	87	255	>80	100%	NR	P-MCI	China	5
230	Song et al. (2011)	Cross-sectional	11	88	74–89	44.32%	NR	COMD-3	China	4
231	Xu et al. (2016)	Cross-sectional	24	206	≥75	100%	2012	DSM-IV	China	6
232	Ma et al. (2017)	Cross-sectional	148	895	≥60	48.94%	NR	ADNI	China	5
233	Zhang et al. (2014)	Cross-sectional	287	1,764	≥60	44.05%	2012	Chinese guidelines and P-MCI	China	6

The number of amnestic MCI (aMCI) and no amnestic MCI (naMCI) were reported in these studies.

①NR, not reported; ②P-MCI, classical Petersen’s criteria of MCI; ③DSM, diagnostic and statistical manual of mental disorders; ④ANDI, Alzheimer’s disease neruimaging initiative; ⑤NIA-AA, the National Institute on Aging-Alzheimer’s Association; ⑥CDGM, Chinese guidelines for diagnosis and management of cognitive impairment and dementia; ⑦IWG, international working group; ⑧COMD-3, the spirit of China disorders classification and diagnostic criteria, third edition.

Study quality assessment scores ranged from 4 to 11, with 76 studies (32.6%) rated as “high quality” and 157 studies (67.4%) rated as “moderate quality.” All the 233 studies scored no less than 3, so no study was excluded. Further details of the quality assessment are shown in Supplementary File S3.

### 3.3. Prevalence of MCI

**Figure 2:**
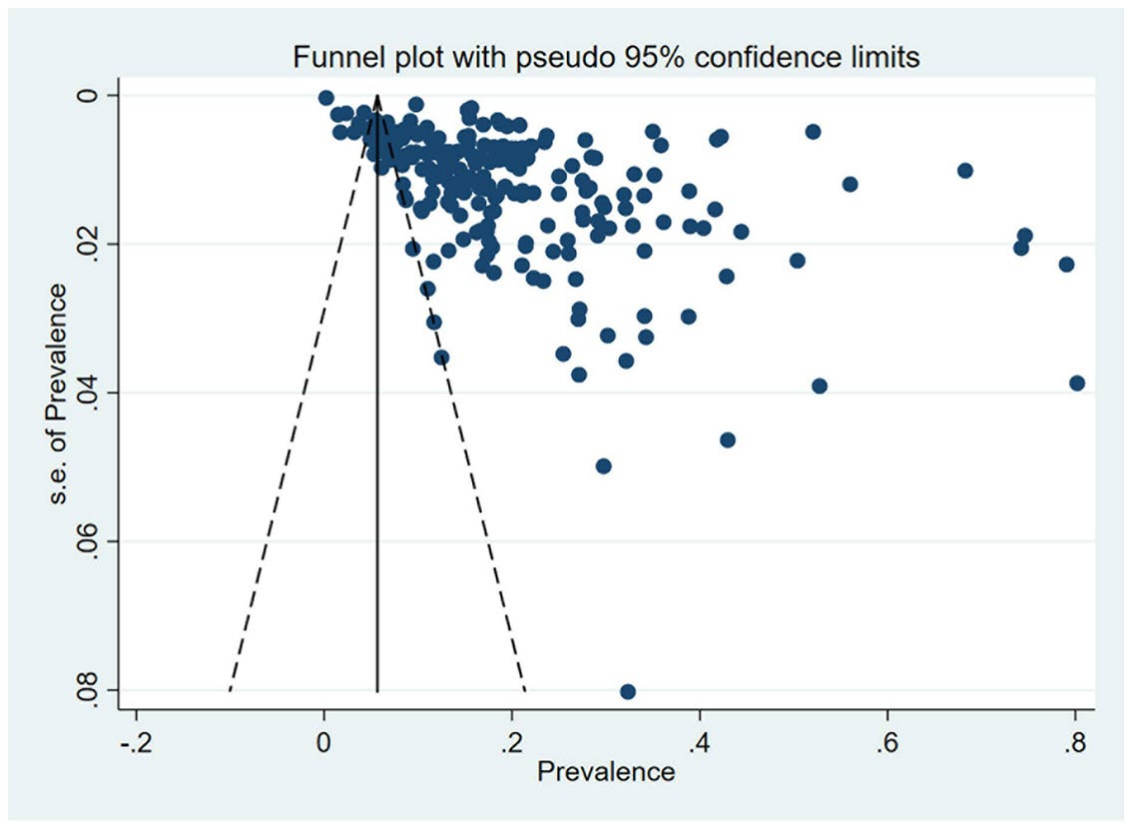
Funnel plot of pooled prevalence of MCI.

A total of 233 studies were included in the analysis of overall pooled prevalence of MCI via a random effect model. The total global prevalence of MCI was 19.7% [(95% CI: 18.3–21.1%), *p-*value^1^ < 0.001, *I^2^* = 99.80%], showing significant heterogeneity among studies. The funnel plot and Egger’s test (*P-*Egger’s test < 0.001) both detected potential publication bias among the pooled results (Figure 2).

### 3.4. Subgroup analyses

Subgroup analyses indicated that the possible sources of heterogeneity were the sample source and beginning year of survey. The total prevalence of MCI in hospitals [34.0% (95% CI: 22.2–45.7%)] was the highest compared to that in nursing homes [22.6% (95% CI: 15.5–29.8%)] and communities [17.9% (95% CI: 16.6–19.2%)]. Moreover, MCI prevalence increased significantly over time. In particular, the global prevalence rose sharply after 2019 [32.1% (95% CI: 22.6–41.6%)] compared to the rates between 2010 and 2018 [19.8% (95% CI: 17.1–22.5%)] and before 2009 [14.5% (95% CI: 12.1–16.9%)]. Subsequently, we conducted further subgroup analyses to explore the time trends in prevalence from different sample sources (Table 2). Surprisingly, there were no significant differences in MCI prevalence among hospitals, nursing homes and communities before 2019. However, the MCI prevalence in hospitals [61.7% (95% CI: 27.8–95.7%)] was significantly higher than in nursing homes [16.1% (95% CI: 14.3–17.9%)] and communities [25.3% (95% CI: 17.4–33.2%)] after 2019. Additionally details of the subgroup analyses can be found in Table 3.

**Table 2:** The time trends in MCI prevalence from different sample sources.

Subgroup	No. of cases	No. of samples	Prevalence, 95%CI (%)	*p*-value^1^	*p*-value^2^
**≤ 2009**					0.228
Community	18,914	106,057	15.8 (13.0–18.6)	<0.001	
Nursing home	356	3,460	13.1 (9.4–16.8)	<0.001	
Hospital	1,513	23,330	35.7 (4.2–67.1)	0.026	
**2010–2018**					0.565
Community	33,245	169,301	18.7 (15.7–21.6)	<0.001	
Nursing home	999	9,438	27.7 (11.4–44.0)	0.001	
Hospital	1,163	6,087	18.8 (13.8–23.8)	<0.001	
**≥ 2019**					0.003
Community	934	5,505	25.3 (17.4–33.2)	<0.001	
Nursing home	260	1,614	16.1 (14.3–17.9)	<0.001	
Hospital	579	1,054	61.7 (27.8–95.7)	<0.001	

*p*-value^1^ is the *p*-value within subgroups; *p*-value^2^ is the *p*-value across subgroups; 95%CI, 95% confidence interval.

**Table 3:** Subgroup analyses of MCI prevalence.

Subgroup	No. study	No. of cases	No. of sample	Prevalence, 95%CI (%)		*p-*value^1^	*p-*value^2^
Overall	233	115,958	676,974	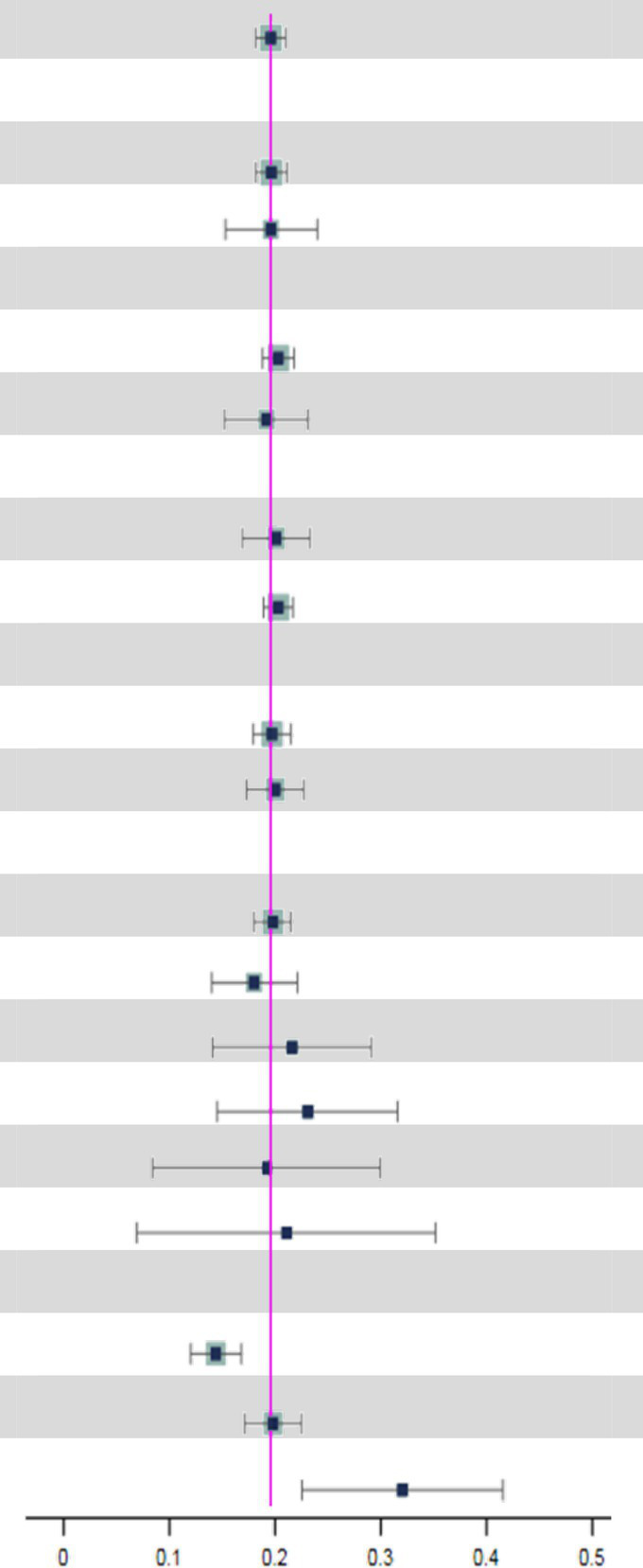	19.7 (18.3–21.1)	<0.001	
Study type							0.976
Cross-sectional	207	106,067	627,798		19.7 (18.2–21.2)	<0.001	
Cohort	26	9,891	49,176		19.6 (15.3–24.0)	<0.001	
Diagnostic method							0.786
P-MCI	150	54,227	309,548		20.1 (18.5–21.6)	<0.001	
DSM	43	34,003	196,537		19.5 (15.7–23.3)	<0.001	
Male to female Ratio							0.918
Male/female ≥1	43	11,351	57,164		20.1 (16.9–23.3)	<0.001	
Male/female <1	164	99,214	560,490		20.3 (18.9–21.7)	<0.001	
Region1							0.856
Developing country	168	66,411	382,725		19.7 (17.9–21.5)	<0.001	
Developed country	60	31,958	182,170		20.0 (17.3–22.7)	<0.001	
Region2							0.909
Asia	171	70,205	400,010		19.8 (18.0–21.5)	<0.001	
Europe	27	20,703	125,743		18.0 (14.0–22.1)	<0.001	
North America	14	4,599	18,936		21.6 (14.1–29.1)	<0.001	
Africa	8	1,251	9,192		23.1 (14.5–31.6)	<0.001	
Oceania	4	713	5,337		19.3 (8.5–30.0)	<0.001	
South America	4	898	5,677		21.2 (7.0–35.3)	0.003	
Beginning year of Survey							<0.001
≤ 2009	54	162,314	20,548		14.5 (12.1–16.9)	<0.001	
2010–2018	72	195,203	40,908		19.8 (17.1–22.5)	<0.001	
≥ 2019	9	2,025	10,024		32.1 (22.6–41.6)	<0.001	
Sample size				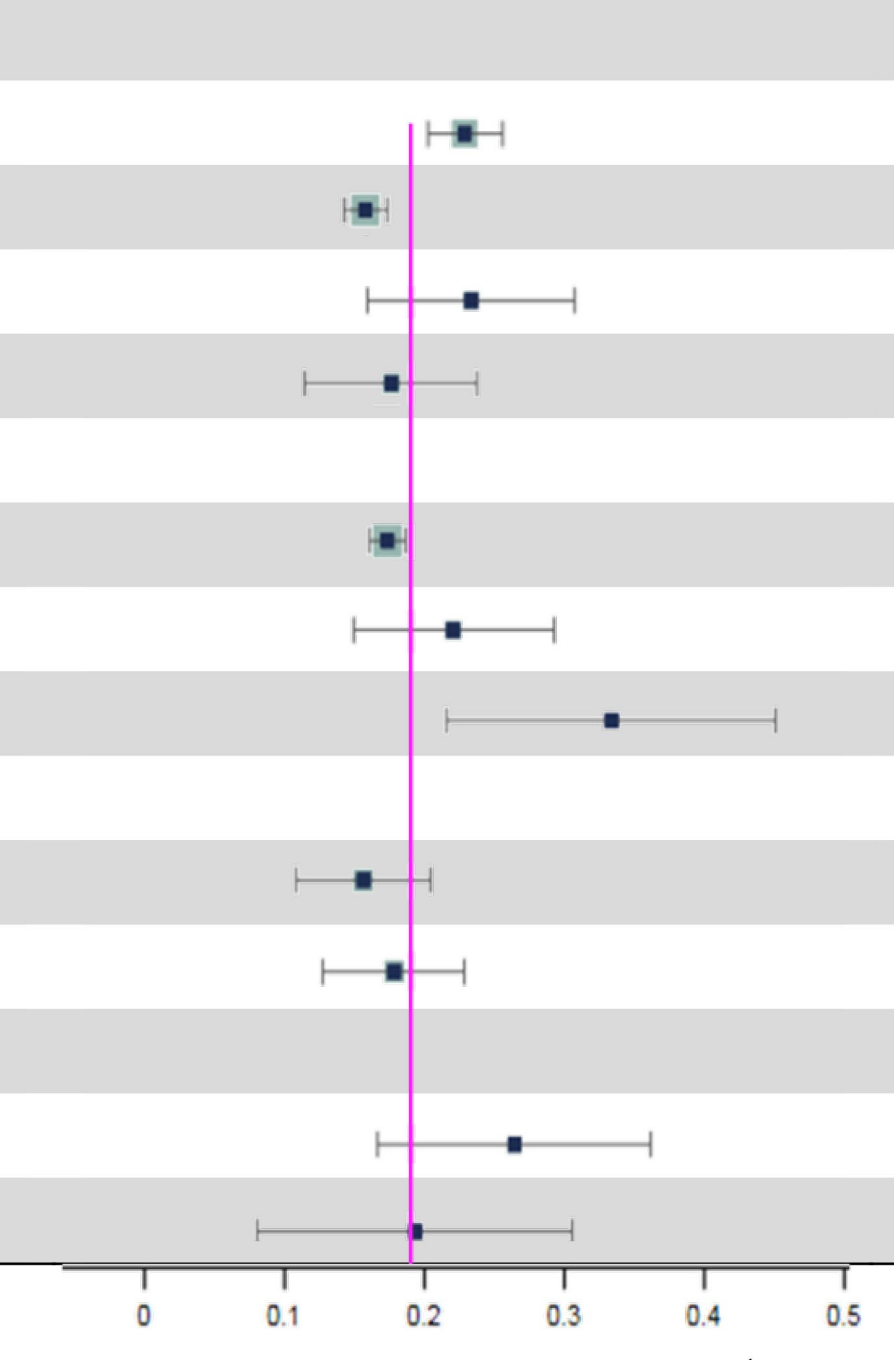			<0.001
0–1,000	94	10,760	48,769		23.5 (20.9–26.2)	<0.001	
1,001–5,000	115	39,651	246,475		16.4 (14.9–18.0)	<0.001	
5,001–10,000	12	21,099	88,648		23.9 (16.5–31.3)	<0.001	
≥10,001	12	44,448	293,082		18.2 (12.0–24.3)	<0.001	
Sample source							0.014
Community	170	84,742	498,057		17.9 (16.6–19.2)	<0.001	
Nursing home	21	8,754	30,251		22.6 (15.5–29.8)	<0.001	
Hospital	16	3,541	31,239		34.0 (22.2–45.7)	<0.001	
MCI subtype							0.555
aMCI/naMCI ≥1	17	7,174	41,589		16.2 (11.4–21.0)	<0.001	
aMCI/naMCI <1	5	1,252	6,535		18.4 (13.3–23.4)	<0.001	
Basic diseases/Non basic diseases							0.349
≥ 1	7	2,026	10,049		27.0 (17.2–36.7)	<0.001	
< 1	6	3,211	15,800		19.9 (8.6–31.1)	0.001	

*p*-value^1^ is the *p*-value within subgroups; *p*-value^2^ is the *p*-value across subgroups; Region^1^ is classified according to developed/developing countries; Region^2^ is based on the region of each country.

①95%CI, 95% confidence interval; ②P-MCI, classical Petersen’s criteria of MCI; ③DSM, diagnostic and statistical manual of mental disorders; ④MCI, mild cognitive impairment.

## 4. Discussion

Previous studies revealed partial results when investigating the prevalence of MCI with different degrees of limitation. In our study, we conducted an extensive literature search based on seven electronic databases and manual retrieval, ultimately identifying 233 studies with a total of 115,958 participants. Furthermore, we included more variables of interest into subgroup analyses, such as sample source, basic diseases, the beginning year of survey, and others. Considering the COVID-19 pandemic period, we attached importance to the MCI prevalence before and after 2019. To our knowledge, this is the most recent meta-analysis to provide a comprehensive overview of MCI prevalence without any limitations in age or region.

We concluded that the global total prevalence of MCI is 19.7% (95% CI: 18.3–21.1%) among 233 included studies. In addition, Subgroup analyses revealed that the sample source and beginning year of survey were considered factors potentially associated with MCI prevalence (*p-*value^2^ < 0.05) (Table 3).

On the one hand, the prevalence of MCI patients in hospitals [34.0% (95% CI: 22.2–45.7%)] was higher than those in nursing homes [22.6% (95% CI: 15.5–29.8%)] and communities [17.9% (95% CI: 16.6–19.2%)]. Several previous studies also draw the consistent conclusions. For example, Xue et al. reported that clinical patients [16.72% (95% CI: 15.6–17.7%)] have a higher MCI prevalence than nonclinical patients [14.61% (95% CI: 14.4–14.8%)] (Xue et al., 2018). The higher MCI prevalence in hospitals may be attributed to professional diagnosis and treatment procedures. Meanwhile, patients in hospitals have more apparent clinical symptoms of MCI and receive more attention from clinicians, which greatly improves the detection rate of MCI. Similarly, the population in nursing homes [21.2% (95% CI: 18.7–23.6%)] have a higher MCI prevalence than community dwellers [5.56% (95% CI: 13.2–18.0%)] (Bai et al., 2022; Chen et al., 2023). Compared to those living in nursing homes, people living in the communities have better material and emotional support from their families, which might make a difference in reducing MCI prevalence.

On the other hand, we found that the total prevalence of MCI increased over time, especially after 2019. Notably, before 2019, there were no significant differences in MCI prevalence among three sample sources. However, the MCI prevalence after 2019 in hospitals [61.7% (95% CI: 27.8–95.7%)] was significantly higher than those in nursing homes [16.1% (95% CI: 14.3–17.9%)] and communities [25.3% (95% CI: 17.4–33.2%)] (Table 2). Since the COVID-19 outbreak globally in 2019, hospital with the support of limited health resources and medical personnel with professional clinical knowledge has become the main refuge for COVID-19 patients (Kadri et al., 2020; Wadhera et al., 2020). There is cumulative evidence suggesting that COVID-19 impacts brain function and is associated with an elevated risk of neurodegenerative conditions, including cognitive dysfunction (Miners et al., 2020; Nath, 2020; Alquisiras-Burgos et al., 2021). Various post-COVID-19 symptoms indicate that coronaviruses, including SARS-CoV-2, could infect the central nervous system (CNS) through hematogenous pathways or neuronal retrograde neuro-invasion. This infiltration leads to subsequent microglial activation and enduring neuroinflammation, with dysregulated neuro-immunity serving as a foundational cause of nerve cell damage (Ellul et al., 2020; Troyer et al., 2020). Supporting the theory that COVID-19 can influence and exacerbate cognitive dysfunction, our data reveals a notable spike in the prevalence of MCI in hospitals post-2019. However, this rate may be conservative. The causes for this speculation are likely multifactorial, such as patients avoidance of emergency care due to fear of COVID-19 or the increased threshold for hospitalization of non-COVID-19 patients by clinicians due to the severity and urgency of COVID-19 (Blecker et al., 2021), which could masks the true prevalence. Therefore, more studies are needed in the future to investigate the potential link between COVID-19 and MCI.

## 5. Strengths and limitations

Based on previous research, this meta-analysis is the latest meta-analysis to provide a comprehensive overview of MCI prevalence without any age and regional limitations. This meta-analysis may aid policymakers, clinicians in making decisions and clinical directions, thus facilitating future studies and clinical applications. Our study, including the most extensive information currently available, is the first to analyze the association between COVID-19 and global MCI prevalence. However, there are also some limitations. First, the included data is unevenly distributed across regions. A large number of studies have been included from Asia, Europe, and North America, while relatively few have been included from Africa, Oceania, and South America. This unbalanced distribution of literature across regions may introduce bias in subgroups. Naturally, due to the vast amount of data included, our study unavoidably presents significant publication bias. Finally, the MCI prevalence in post-COVID-19 era still requires further investigation to provide more accurate evidence for the allocation of medical and health resources.

## 6. Conclusion

Our systematic review indicates that the current pooled global prevalence of Mild Cognitive Impairment (MCI) stands at 19.7%. Notably, we found a significant correlation between beginning year of survey and the global prevalence of MCI, with prevalence rates rising significantly after 2019. Furthermore, it is noteworthy that the prevalence of MCI in hospital settings outstripped those in nursing homes and community settings, especially after 2019. This trend may be in part attributable to the outbreak of COVID-19. The potential connection between COVID-19 and MCI warrants further investigation in future studies. Lastly, we posit that our review holds substantial value for policymakers and clinicians. The insights gleaned can guide health-related decision-making processes and inform the strategic allocation of health resources to better serve patients with MCI.

## Data availability statement

The original contributions presented in the study are included in the article/Supplementary material, further inquiries can be directed to the corresponding authors.

## Author contributions

J-hL, JC, and S-xX conceived and designed the study. W-xS and W-wW performed the data analysis and wrote the manuscript. Y-yZ, H-lX, S-yJ, and G-cC assessed the literature and extracted the data. All authors contributed to the article and approved the submitted version.
