# Supplementary material for: Evidence from a meta-analysis and systematic review reveals the global prevalence of mild cognitive impairment
Source: Front Aging Neurosci. 2023 Oct 27;15:1227112. doi: 10.3389/fnagi.2023.1227112 (PMC10641463; doi:10.3389/fnagi.2023.1227112)
Supplement: Supplementary file 1 [file Data_Sheet_1.docx]

**Search strategies**

**PubMed**

1. "Cognitive Dysfunction"[Mesh]
2. ((((((((((((((((((((((((((('Cognitive Decline'[Title/Abstract]) OR ('Cognitive Declines'[Title/Abstract])) OR ('Cognitive Disorder'[Title/Abstract])) OR ('Cognitive Disorders'[Title/Abstract])) OR ('Mild Cognitive Disorders'[Title/Abstract])) OR (Mild Cognitive Disorder[Title/Abstract])) OR ('Cognitive Dysfunction'[Title/Abstract])) OR ('Cognitive Dysfunctions'[Title/Abstract])) OR ('Cognitive Impairment'[Title/Abstract])) OR ('Cognitive Impairments'[Title/Abstract])) OR ('Mild Cognitive Impairment'[Title/Abstract])) OR ('Mild Cognitive Impairments'[Title/Abstract])) OR ('Mental Deterioration'[Title/Abstract])) OR ('Mental Deteriorations'[Title/Abstract])) OR ('Dysfunction, Cognitive'[Title/Abstract])) OR ('Dysfunctions, Cognitive'[Title/Abstract])) OR ('Impairment, Cognitive'[Title/Abstract])) OR ('Impairments, Cognitive'[Title/Abstract])) OR ('Disorder, Cognitive'[Title/Abstract])) OR ('Disorders, Cognitive'[Title/Abstract])) OR ('Cognitive Impairment, Mild'[Title/Abstract])) OR ('Cognitive Impairments, Mild'[Title/Abstract])) OR ('Impairment, Mild Cognitive'[Title/Abstract])) OR ('Impairments, Mild Cognitive'[Title/Abstract])) OR ('Decline, Cognitive'[Title/Abstract])) OR ('Declines, Cognitive'[Title/Abstract])) OR ('Deterioration, Mental'[Title/Abstract])) OR ('Deteriorations, Mental'[Title/Abstract])
3. ("Cognitive Dysfunction"[Mesh]) OR (((((((((((((((((((((((((((('Cognitive Decline'[Title/Abstract]) OR ('Cognitive Declines'[Title/Abstract])) OR ('Cognitive Disorder'[Title/Abstract])) OR ('Cognitive Disorders'[Title/Abstract])) OR ('Mild Cognitive Disorders'[Title/Abstract])) OR (Mild Cognitive Disorder[Title/Abstract])) OR ('Cognitive Dysfunction'[Title/Abstract])) OR ('Cognitive Dysfunctions'[Title/Abstract])) OR ('Cognitive Impairment'[Title/Abstract])) OR ('Cognitive Impairments'[Title/Abstract])) OR ('Mild Cognitive Impairment'[Title/Abstract])) OR ('Mild Cognitive Impairments'[Title/Abstract])) OR ('Mental Deterioration'[Title/Abstract])) OR ('Mental Deteriorations'[Title/Abstract])) OR ('Dysfunction, Cognitive'[Title/Abstract])) OR ('Dysfunctions, Cognitive'[Title/Abstract])) OR ('Impairment, Cognitive'[Title/Abstract])) OR ('Impairments, Cognitive'[Title/Abstract])) OR ('Disorder, Cognitive'[Title/Abstract])) OR ('Disorders, Cognitive'[Title/Abstract])) OR ('Cognitive Impairment, Mild'[Title/Abstract])) OR ('Cognitive Impairments, Mild'[Title/Abstract])) OR ('Impairment, Mild Cognitive'[Title/Abstract])) OR ('Impairments, Mild Cognitive'[Title/Abstract])) OR ('Decline, Cognitive'[Title/Abstract])) OR ('Declines, Cognitive'[Title/Abstract])) OR ('Deterioration, Mental'[Title/Abstract])) OR ('Deteriorations, Mental'[Title/Abstract]))
4. "Prevalence"[Mesh]
5. ((((((('prevalence'[Title/Abstract]) OR ('Prevalences'[Title/Abstract])) OR ('Period Prevalence'[Title/Abstract])) OR ('Period Prevalences'[Title/Abstract])) OR ('Prevalence, Period'[Title/Abstract])) OR ('Point Prevalence'[Title/Abstract])) OR ('Point Prevalences'[Title/Abstract])) OR ('Prevalence, Point'[Title/Abstract])
6. ("Prevalence"[Mesh]) OR (((((((('prevalence'[Title/Abstract]) OR ('Prevalences'[Title/Abstract])) OR ('Period Prevalence'[Title/Abstract])) OR ('Period Prevalences'[Title/Abstract])) OR ('Prevalence, Period'[Title/Abstract])) OR ('Point Prevalence'[Title/Abstract])) OR ('Point Prevalences'[Title/Abstract])) OR ('Prevalence, Point'[Title/Abstract]))
7. "Epidemiology"[Mesh]
8. ((((((((((((((('Epidemiology'[Title/Abstract]) OR ('Epidemiologies'[Title/Abstract])) OR ('Epidemiologic Studies'[Title/Abstract])) OR ('Epidemiological Studies'[Title/Abstract])) OR ('Epidemiological Study'[Title/Abstract])) OR ('Epidemiologic Study'[Title/Abstract])) OR ('Studies, Epidemiological'[Title/Abstract])) OR ('Study, Epidemiological'[Title/Abstract])) OR ('Studies, Epidemiologic'[Title/Abstract])) OR ('Study, Epidemiologic'[Title/Abstract])) OR ('Social Epidemiology'[Title/Abstract])) OR ('Social Epidemiologies'[Title/Abstract])) OR ('Epidemiologies, Social'[Title/Abstract])) OR ('Epidemiology, Social'[Title/Abstract])) OR ('Epidemiologic'[Title/Abstract])) OR ('Epidemiological'[Title/Abstract])
9. ("Epidemiology"[Mesh]) OR (((((((((((((((('Epidemiology'[Title/Abstract]) OR ('Epidemiologies'[Title/Abstract])) OR ('Epidemiologic Studies'[Title/Abstract])) OR ('Epidemiological Studies'[Title/Abstract])) OR ('Epidemiological Study'[Title/Abstract])) OR ('Epidemiologic Study'[Title/Abstract])) OR ('Studies, Epidemiological'[Title/Abstract])) OR ('Study, Epidemiological'[Title/Abstract])) OR ('Studies, Epidemiologic'[Title/Abstract])) OR ('Study, Epidemiologic'[Title/Abstract])) OR ('Social Epidemiology'[Title/Abstract])) OR ('Social Epidemiologies'[Title/Abstract])) OR ('Epidemiologies, Social'[Title/Abstract])) OR ('Epidemiology, Social'[Title/Abstract])) OR ('Epidemiologic'[Title/Abstract])) OR ('Epidemiological'[Title/Abstract]))
10. (("Prevalence"[Mesh]) OR (((((((('prevalence'[Title/Abstract]) OR ('Prevalences'[Title/Abstract])) OR ('Period Prevalence'[Title/Abstract])) OR ('Period Prevalences'[Title/Abstract])) OR ('Prevalence, Period'[Title/Abstract])) OR ('Point Prevalence'[Title/Abstract])) OR ('Point Prevalences'[Title/Abstract])) OR ('Prevalence, Point'[Title/Abstract]))) OR (("Epidemiology"[Mesh]) OR (((((((((((((((('Epidemiology'[Title/Abstract]) OR ('Epidemiologies'[Title/Abstract])) OR ('Epidemiologic Studies'[Title/Abstract])) OR ('Epidemiological Studies'[Title/Abstract])) OR ('Epidemiological Study'[Title/Abstract])) OR ('Epidemiologic Study'[Title/Abstract])) OR ('Studies, Epidemiological'[Title/Abstract])) OR ('Study, Epidemiological'[Title/Abstract])) OR ('Studies, Epidemiologic'[Title/Abstract])) OR ('Study, Epidemiologic'[Title/Abstract])) OR ('Social Epidemiology'[Title/Abstract])) OR ('Social Epidemiologies'[Title/Abstract])) OR ('Epidemiologies, Social'[Title/Abstract])) OR ('Epidemiology, Social'[Title/Abstract])) OR ('Epidemiologic'[Title/Abstract])) OR ('Epidemiological'[Title/Abstract])))
11. (("Cognitive Dysfunction"[Mesh]) OR (((((((((((((((((((((((((((('Cognitive Decline'[Title/Abstract]) OR ('Cognitive Declines'[Title/Abstract])) OR ('Cognitive Disorder'[Title/Abstract])) OR ('Cognitive Disorders'[Title/Abstract])) OR ('Mild Cognitive Disorders'[Title/Abstract])) OR (Mild Cognitive Disorder[Title/Abstract])) OR ('Cognitive Dysfunction'[Title/Abstract])) OR ('Cognitive Dysfunctions'[Title/Abstract])) OR ('Cognitive Impairment'[Title/Abstract])) OR ('Cognitive Impairments'[Title/Abstract])) OR ('Mild Cognitive Impairment'[Title/Abstract])) OR ('Mild Cognitive Impairments'[Title/Abstract])) OR ('Mental Deterioration'[Title/Abstract])) OR ('Mental Deteriorations'[Title/Abstract])) OR ('Dysfunction, Cognitive'[Title/Abstract])) OR ('Dysfunctions, Cognitive'[Title/Abstract])) OR ('Impairment, Cognitive'[Title/Abstract])) OR ('Impairments, Cognitive'[Title/Abstract])) OR ('Disorder, Cognitive'[Title/Abstract])) OR ('Disorders, Cognitive'[Title/Abstract])) OR ('Cognitive Impairment, Mild'[Title/Abstract])) OR ('Cognitive Impairments, Mild'[Title/Abstract])) OR ('Impairment, Mild Cognitive'[Title/Abstract])) OR ('Impairments, Mild Cognitive'[Title/Abstract])) OR ('Decline, Cognitive'[Title/Abstract])) OR ('Declines, Cognitive'[Title/Abstract])) OR ('Deterioration, Mental'[Title/Abstract])) OR ('Deteriorations, Mental'[Title/Abstract]))) AND ((("Prevalence"[Mesh]) OR (((((((('prevalence'[Title/Abstract]) OR ('Prevalences'[Title/Abstract])) OR ('Period Prevalence'[Title/Abstract])) OR ('Period Prevalences'[Title/Abstract])) OR ('Prevalence, Period'[Title/Abstract])) OR ('Point Prevalence'[Title/Abstract])) OR ('Point Prevalences'[Title/Abstract])) OR ('Prevalence, Point'[Title/Abstract]))) OR (("Epidemiology"[Mesh]) OR (((((((((((((((('Epidemiology'[Title/Abstract]) OR ('Epidemiologies'[Title/Abstract])) OR ('Epidemiologic Studies'[Title/Abstract])) OR ('Epidemiological Studies'[Title/Abstract])) OR ('Epidemiological Study'[Title/Abstract])) OR ('Epidemiologic Study'[Title/Abstract])) OR ('Studies, Epidemiological'[Title/Abstract])) OR ('Study, Epidemiological'[Title/Abstract])) OR ('Studies, Epidemiologic'[Title/Abstract])) OR ('Study, Epidemiologic'[Title/Abstract])) OR ('Social Epidemiology'[Title/Abstract])) OR ('Social Epidemiologies'[Title/Abstract])) OR ('Epidemiologies, Social'[Title/Abstract])) OR ('Epidemiology, Social'[Title/Abstract])) OR ('Epidemiologic'[Title/Abstract])) OR ('Epidemiological'[Title/Abstract]))))

**Embase**

1. # 'mild cognitive impairment'/exp OR 'cognitive declines':ab,ti OR 'cognitive declines':ab,ti OR 'cognitive disorder':ab,ti OR 'cognitive disorders':ab,ti OR 'mild cognitive disorders':ab,ti OR 'mild cognitive disorder':ab,ti OR 'cognitive dysfunction':ab,ti OR 'cognitive dysfunctions':ab,ti OR 'cognitive impairment':ab,ti OR 'cognitive impairments':ab,ti OR 'mild cognitive impairment':ab,ti OR 'mild cognitive impairments':ab,ti OR 'mental deterioration':ab,ti OR 'mental deteriorations':ab,ti OR 'dysfunction, cognitive':ab,ti OR 'dysfunctions, cognitive':ab,ti OR 'impairment, cognitive':ab,ti OR 'impairments, cognitive':ab,ti OR 'disorder, cognitive':ab,ti OR 'disorders, cognitive':ab,ti OR 'cognitive impairment, mild':ab,ti OR 'cognitive impairments, mild':ab,ti OR 'impairment, mild cognitive':ab,ti OR 'impairments, mild cognitive':ab,ti OR 'decline, cognitive':ab,t OR 'declines, cognitive':ab,ti OR 'deterioration, mental':ab,t OR 'deteriorations, mental':ab,ti
2. 'prevalence'/exp OR 'prevalence':ab,ti OR 'prevalences':ab,ti OR 'period prevalence':ab,ti OR 'period prevalences':ab,ti OR 'prevalence, period':ab,t OR 'point prevalence':ab,ti OR 'point prevalences':ab,ti OR 'prevalence, point':ab,ti
3. 'epidemiology'/exp OR 'epidemiology':ab,ti OR 'epidemiologies':ab,ti OR 'epidemiologic studies':ab,ti OR 'epidemiological studies':ab,ti OR 'epidemiological study':ab,ti OR'epidemiologic study':ab,ti OR 'studies, epidemiological':ab,ti OR 'study, epidemiological':ab,ti OR 'studies, epidemiologic':ab,ti OR 'study, epidemiologic':ab,ti OR 'social epidemiology':ab,ti OR 'social epidemiologies':ab,ti OR 'epidemiologies, social':ab,ti OR 'epidemiology, social':ab,ti OR #'epidemiologic':ab,ti OR 'epidemiological':ab,ti
4. #2 OR #3
5. #1 AND #4

**Web of Science (WOS)**

TS=(‘Cognitive Decline’ OR ‘Cognitive Declines’ OR ‘Cognitive Disorder’ OR ‘Cognitive Disorders’ OR ‘Mild Cognitive Disorders’ OR ‘Mild Cognitive Disorder’ OR ‘Cognitive Dysfunction’ OR ‘Cognitive Dysfunctions’ OR ‘Cognitive Impairment’ OR ‘Cognitive Impairments’ OR ‘Mild Cognitive Impairment’ OR ‘Mild Cognitive Impairments’ OR ‘Mental Deterioration’ OR ‘Mental Deteriorations’ OR ‘Dysfunction, Cognitive’ OR ‘Dysfunctions, Cognitive’ OR ‘Impairment, Cognitive' OR 'Impairments, Cognitive’ OR ‘Disorder, Cognitive’ OR ‘Disorders, Cognitive’ OR ‘Cognitive Impairment, Mild’ OR ‘Cognitive Impairments, Mild’ OR ‘Impairment, Mild Cognitive’ OR ‘Impairments, Mild Cognitive’ OR ‘Decline, Cognitive’ OR ‘Declines, Cognitive’ OR ‘Deterioration, Mental’ OR ‘Deteriorations, Mental’ OR ‘MCI’)

**Chinese National Knowledge Infrastructure (CNKI)**

( ( ( ( ( ( ( ( ( 主题%= (轻度认知障碍+轻度认知功能障碍+轻度认知功能损伤+轻度认知损伤+轻度认知受损+轻度认知功能受损+轻度认知功能损害+轻度认知损害) or 题名%= (轻度认知障碍+轻度认知功能障碍+轻度认知功能损伤+轻度认知损伤+轻度认知受损+轻度认知功能受损+轻度认知功能损害+轻度认知损害) ) OR ( 主题%= (xls('Cognitive Decline')+xls('Cognitive Declines')+xls('Cognitive Disorder')+xls('Cognitive Disorders')) or 题名%= (xls('Cognitive Decline')+xls('Cognitive Declines')+xls('Cognitive Disorder')+xls('Cognitive Disorders')) ) ) OR ( 主题%= (xls('Cognitive Dysfunction')+xls('Cognitive Dysfunctions')+xls('Cognitive Impairment')+xls('Cognitive Impairments')) or 题名%= (xls('Cognitive Dysfunction')+xls('Cognitive Dysfunctions')+xls('Cognitive Impairment')+xls('Cognitive Impairments')) ) ) OR ( 主题%= (xls('Mild Cognitive Impairment')+xls('Mild Cognitive Impairments')+xls('Mental Deterioration')+xls('Mental Deteriorations')) or 题名%= (xls('Mild Cognitive Impairment')+xls('Mild Cognitive Impairments')+xls('Mental Deterioration')+xls('Mental Deteriorations')) ) ) OR ( 主题%= (xls('Dysfunction, Cognitive')+xls('Dysfunctions, Cognitive')+xls('Impairment, Cognitive')+xls('Impairments, Cognitive')) or 题名%= (xls('Dysfunction, Cognitive')+xls('Dysfunctions, Cognitive')+xls('Impairment, Cognitive')+xls('Impairments, Cognitive')) ) ) OR ( 主题%= (xls('Disorder, Cognitive')+xls('Disorders, Cognitive')+xls('Cognitive Impairment, Mild')+xls('Cognitive Impairments, Mild')) or 题名%= (xls('Disorder, Cognitive')+xls('Disorders, Cognitive')+xls('Cognitive Impairment, Mild')+xls('Cognitive Impairments, Mild')) ) ) OR ( 主题%= (xls('Impairment, Mild Cognitive')+xls('Impairments, Mild Cognitive')+xls('Decline, Cognitive')+xls('Declines, Cognitive')) or 题名%= (xls('Impairment, Mild Cognitive')+xls('Impairments, Mild Cognitive')+xls('Decline, Cognitive')+xls('Declines, Cognitive')) ) ) OR ( 主题%= (xls('Deterioration, Mental')+xls('Deteriorations, Mental')+xls('MCI')) or 题名%= (xls('Deterioration, Mental')+xls('Deteriorations, Mental')+xls('MCI')) ) ) AND ( 主题%= (患病率+流行病学) or 题名%= (患病率+流行病学) ) )

**Wanfang Database (WFD)**

 题名或关键词:(( 轻度认知障碍 or 轻度认知功能障碍 or 轻度认知功能损伤 or 轻度认知损伤 or 轻度认知受损 or 轻度认知功能受损 or 轻度认知功能损害 or 轻度认知损害 )) or 题名或关键词:(( 'Cognitive Decline' or 'Cognitive Disorder' or 'Mild Cognitive Disorder' or 'MCI' )) or 题名或关键词:(( 'Cognitive Dysfunction' or 'Cognitive Impairment' or 'Mild Cognitive Impairment' )) or 题名或关键词:(( 'Mental Deterioration' or 'Dysfunction, Cognitive' or 'Impairment, Cognitive' )) or 题名或关键词:(( 'Disorder, Cognitive' or 'Cognitive Impairment, Mild' or 'Impairment, Mild Cognitive' )) and 题名或关键词:(( 患病率 or 流行病学 ))

**Chinese Biomedical Literature Database (CBM)**

1. "患病率"[不加权:扩展]
2. "患病率"[常用字段:智能] OR "流行病学"[常用字段:智能]
3. (#2) OR (#1)
4. "认知功能障碍"[不加权:扩展]
5. "轻度认知障碍"[常用字段:智能] OR "轻度认知功能障碍"[常用字段:智能] OR "轻度认知功能损伤"[常用字段:智能] OR "轻度认知损伤"[常用字段:智能] OR "轻度认知受损"[常用字段:智能] OR "轻度认知功能受损"[常用字段:智能] OR "轻度认知功能损害"[常用字段:智能] OR "轻度认知损害"[常用字段:智能] OR "'MCI'"[常用字段:智能]
6. (#5) OR (#4)
7. (#6) AND (#3)

**China Science and Technology Journal Database (VIP)**

( M = ( 轻度认知障碍 OR 轻度认知功能障碍 OR 轻度认知功能损伤 OR 轻度认知损伤 OR 轻度认知受损 OR 轻度认知功能受损 OR 轻度认知功能损害 OR 轻度认知损害 ) OR M = ( 'Cognitive Decline' OR 'Cognitive Declines' OR 'Cognitive Disorder' OR 'Cognitive Disorders' OR 'Mild Cognitive Disorders' OR 'Mild Cognitive Disorder' OR 'Cognitive Dysfunction' OR 'Cognitive Dysfunctions' OR 'Cognitive Impairment' OR 'Cognitive Impairments' OR 'Mild Cognitive Impairment ' OR 'Mild Cognitive Impairments' OR 'Mental Deterioration' OR 'Mental Deteriorations' OR 'Dysfunction, Cognitive' OR 'Dysfunctions, Cognitive' OR 'Impairment, Cognitive' OR 'Impairments, Cognitive' OR 'Disorder, Cognitive' OR 'Disorders, Cognitive' OR 'Cognitive Impairment, Mild' OR 'Cognitive Impairments, Mild' OR 'Impairment, Mild Cognitive' OR 'Impairments, Mild Cognitive' OR 'Decline, Cognitive' OR 'Declines, Cognitive' OR 'Deterioration, Mental' OR 'Deteriorations, Mental' OR 'MCI' ) ) AND ( M = ( 患病率 OR 流行病学 ) )
