# Supplementary material for: Evidence from a meta-analysis and systematic review reveals the global prevalence of mild cognitive impairment
Source: Front Aging Neurosci. 2023 Oct 27;15:1227112. doi: 10.3389/fnagi.2023.1227112 (PMC10641463; doi:10.3389/fnagi.2023.1227112)
Supplement: Supplementary file 2 [file Data_Sheet_2.docx]

**Supplementary File 2. Quality assessment of the literature for MCI prevalence inclusion: observation cohort and cross-Sectional studies**

| ID | Study | Ⅰ | Ⅱ | Ⅲ | Ⅳ | Ⅴ | Ⅵ | Ⅶ | Ⅷ | Ⅸ | Ⅹ | Ⅺ | Summary |
| --- | --- | --- | --- | --- | --- | --- | --- | --- | --- | --- | --- | --- | --- |
| 1 | Björk et al. 2018 | 1 | 1 | 1 | 1 | 1 | 1 | 2 | 1 | 1 | 1 | 2 | 9 |
| 2 | Tiwari et al. 2013 | 1 | 1 | 1 | 1 | 1 | 1 | 1 | 1 | 3 | 2 | 3 | 8 |
| 3 | Rao et al. 2018 | 1 | 1 | 1 | 1 | 1 | 1 | 3 | 1 | 2 | 1 | 3 | 8 |
| 4 | VancamPfort et al. 2017 | 1 | 1 | 1 | 1 | 1 | 1 | 2 | 1 | 2 | 2 | 3 | 7 |
| 5 | Lu et al. 2019 | 1 | 1 | 1 | 1 | 2 | 1 | 1 | 1 | 2 | 2 | 3 | 7 |
| 6 | Su et al. 2013 | 1 | 2 | 1 | 1 | 2 | 1 | 2 | 2 | 3 | 2 | 2 | 4 |
| 7 | Zhang et al. 2013 | 1 | 1 | 1 | 1 | 2 | 1 | 2 | 2 | 3 | 2 | 2 | 5 |
| 8 | Zhang et al. 2015 | 1 | 1 | 2 | 1 | 3 | 1 | 2 | 1 | 2 | 1 | 2 | 6 |
| 9 | Li et al. 2013 | 1 | 1 | 1 | 1 | 1 | 1 | 1 | 1 | 3 | 1 | 2 | 9 |
| 10 | Guo et al. 2013 | 1 | 1 | 3 | 1 | 1 | 1 | 2 | 1 | 3 | 2 | 2 | 6 |
| 11 | Yin et al. 2012 | 1 | 1 | 1 | 1 | 1 | 1 | 2 | 1 | 3 | 2 | 2 | 7 |
| 12 | Pan et al. 2012 | 1 | 1 | 1 | 1 | 1 | 1 | 1 | 1 | 3 | 1 | 2 | 9 |
| 13 | Xia et al. 2006 | 1 | 1 | 1 | 1 | 1 | 1 | 2 | 1 | 3 | 2 | 2 | 7 |
| 14 | Yang et al. 2017 | 1 | 1 | 3 | 3 | 1 | 1 | 1 | 1 | 1 | 2 | 3 | 7 |
| 15 | Zhang et al. 2018 | 1 | 1 | 1 | 1 | 1 | 1 | 2 | 2 | 3 | 1 | 2 | 7 |
| 16 | Jiang et al. 2019 | 1 | 1 | 3 | 1 | 1 | 1 | 1 | 3 | 3 | 1 | 2 | 7 |
| 17 | Dai et al. 2019 | 1 | 1 | 1 | 1 | 1 | 1 | 2 | 1 | 3 | 1 | 2 | 8 |
| 18 | Liu et al. 2019 | 1 | 1 | 1 | 1 | 1 | 1 | 1 | 1 | 3 | 1 | 2 | 9 |
| 19 | Yuan et al. 2019 | 1 | 1 | 1 | 1 | 1 | 1 | 2 | 3 | 3 | 2 | 2 | 6 |
| 20 | Yuan et al. 2021 | 1 | 1 | 1 | 1 | 1 | 1 | 2 | 1 | 3 | 1 | 2 | 8 |
| 21 | Luo et al. 2015 | 1 | 1 | 1 | 1 | 3 | 1 | 1 | 3 | 2 | 2 | 2 | 6 |
| 22 | Xu et al. 2014 | 1 | 1 | 1 | 1 | 1 | 1 | 2 | 1 | 3 | 1 | 2 | 8 |
| 23 | Tang et al. 2007 | 1 | 2 | 1 | 1 | 1 | 1 | 2 | 1 | 3 | 2 | 2 | 6 |
| 24 | Gang et al. 2008 | 1 | 1 | 1 | 1 | 1 | 1 | 2 | 1 | 3 | 1 | 2 | 8 |
| 25 | Huang et al. 2008 | 1 | 1 | 1 | 1 | 1 | 1 | 2 | 1 | 3 | 1 | 2 | 8 |
| 26 | Ren et al. 2013 | 1 | 1 | 1 | 1 | 1 | 1 | 2 | 1 | 3 | 1 | 2 | 8 |
| 27 | Zhou et al. 2011 | 1 | 1 | 1 | 1 | 1 | 1 | 1 | 1 | 3 | 3 | 2 | 8 |
| 28 | Chen et al. 2015 | 1 | 2 | 2 | 1 | 1 | 1 | 2 | 2 | 2 | 2 | 3 | 4 |
| 29 | Pan et al. 2012 | 1 | 1 | 2 | 1 | 1 | 1 | 1 | 1 | 2 | 2 | 2 | 7 |
| 30 | Song et al. 2012 | 1 | 1 | 1 | 1 | 1 | 1 | 2 | 1 | 2 | 1 | 3 | 8 |
| 31 | Zhu et al. 2009 | 1 | 1 | 1 | 1 | 1 | 1 | 1 | 1 | 2 | 2 | 3 | 8 |
| 32 | Wu et al. 2012 | 1 | 2 | 1 | 1 | 1 | 1 | 2 | 1 | 2 | 1 | 3 | 7 |
| 33 | Liao et al. 2012 | 1 | 2 | 2 | 1 | 1 | 1 | 2 | 1 | 2 | 2 | 2 | 5 |
| 34 | Zhang et al. 2014 | 1 | 1 | 1 | 1 | 1 | 1 | 2 | 1 | 2 | 2 | 3 | 7 |
| 35 | Afgin et al. 2012 | 1 | 1 | 1 | 1 | 1 | 1 | 1 | 1 | 3 | 1 | 1 | 10 |
| 36 | Artero et al. 2008 | 1 | 1 | 1 | 1 | 1 | 1 | 2 | 1 | 2 | 1 | 1 | 9 |
| 37 | Lee et al. 2009 | 1 | 1 | 1 | 1 | 1 | 1 | 2 | 2 | 2 | 1 | 1 | 8 |
| 38 | Ogunniyi et al. 2016 | 1 | 1 | 1 | 1 | 3 | 1 | 2 | 1 | 1 | 1 | 1 | 9 |
| 39 | Petersen et al. 2010 | 1 | 1 | 1 | 1 | 1 | 1 | 1 | 1 | 1 | 1 | 1 | 11 |
| 40 | Pilleron et al. 2015 | 1 | 1 | 1 | 1 | 1 | 1 | 2 | 2 | 2 | 2 | 3 | 6 |
| 41 | Richard et al. 2013 | 1 | 1 | 1 | 1 | 2 | 1 | 2 | 1 | 2 | 1 | 1 | 8 |
| 42 | Kumar et al. 2005 | 1 | 3 | 1 | 1 | 1 | 1 | 1 | 3 | 1 | 1 | 1 | 9 |
| 43 | Lee et al. 2009 | 1 | 3 | 1 | 1 | 1 | 1 | 2 | 3 | 3 | 1 | 3 | 6 |
| 44 | Lee et al. 2012 | 1 | 1 | 1 | 1 | 1 | 1 | 1 | 3 | 3 | 1 | 2 | 8 |
| 45 | Purser et al. 2005 | 1 | 1 | 1 | 1 | 1 | 1 | 1 | 1 | 3 | 2 | 3 | 8 |
| 46 | De Jager et al. 2005 | 1 | 1 | 1 | 1 | 3 | 1 | 1 | 3 | 3 | 2 | 1 | 7 |
| 47 | Khedr et al. 2015 | 1 | 1 | 1 | 1 | 1 | 1 | 3 | 1 | 3 | 3 | 3 | 7 |
| 48 | Yu et al. 2016 | 1 | 3 | 3 | 3 | 1 | 1 | 1 | 1 | 2 | 2 | 3 | 5 |
| 49 | Ma et al. 2016 | 1 | 1 | 1 | 1 | 1 | 1 | 1 | 1 | 3 | 1 | 2 | 9 |
| 50 | Wang et al. 2015 | 1 | 1 | 1 | 1 | 1 | 1 | 1 | 1 | 3 | 2 | 2 | 8 |
| 51 | Jia et al. 2013 | 1 | 1 | 1 | 1 | 1 | 1 | 1 | 1 | 1 | 2 | 3 | 9 |
| 52 | Hu et al. 2012 | 1 | 2 | 1 | 1 | 1 | 1 | 3 | 1 | 3 | 1 | 3 | 7 |
| 53 | Qiu et al. 2003 | 1 | 1 | 1 | 1 | 3 | 1 | 1 | 1 | 3 | 1 | 2 | 8 |
| 54 | Lei et al. 2008 | 1 | 1 | 1 | 1 | 1 | 1 | 1 | 1 | 3 | 2 | 3 | 8 |
| 55 | Lao et al. 2011 | 1 | 3 | 1 | 1 | 3 | 1 | 2 | 1 | 2 | 2 | 3 | 5 |
| 56 | Yang et al. 2011 | 1 | 1 | 1 | 2 | 3 | 1 | 2 | 2 | 2 | 2 | 2 | 4 |
| 57 | Yin et al. 2011 | 1 | 1 | 1 | 1 | 3 | 1 | 2 | 1 | 2 | 2 | 3 | 6 |
| 58 | Tong et al. 2013 | 1 | 1 | 1 | 1 | 1 | 1 | 3 | 2 | 3 | 2 | 2 | 6 |
| 59 | Xiong et al. 2013 | 1 | 1 | 1 | 1 | 1 | 1 | 1 | 2 | 3 | 2 | 3 | 7 |
| 60 | Zhang et al. 2013 | 1 | 1 | 1 | 1 | 1 | 1 | 1 | 2 | 3 | 1 | 2 | 8 |
| 61 | Gu et al. 2014 | 1 | 1 | 1 | 3 | 3 | 1 | 1 | 2 | 1 | 1 | 2 | 7 |
| 62 | Qin et al. 2014 | 1 | 1 | 1 | 1 | 1 | 1 | 2 | 2 | 1 | 1 | 2 | 8 |
| 63 | Sun et al. 2016 | 1 | 2 | 1 | 1 | 2 | 1 | 2 | 3 | 2 | 2 | 2 | 4 |
| 64 | Zhou et al. 2016 | 1 | 1 | 1 | 1 | 1 | 1 | 2 | 1 | 2 | 2 | 2 | 7 |
| 65 | Guo et al. 2012 | 1 | 1 | 1 | 1 | 1 | 1 | 1 | 1 | 2 | 2 | 2 | 8 |
| 66 | Jia et al. 2014 | 1 | 1 | 1 | 1 | 1 | 1 | 2 | 1 | 3 | 1 | 3 | 8 |
| 67 | Li et al. 2013 | 1 | 1 | 1 | 1 | 1 | 1 | 1 | 1 | 2 | 2 | 3 | 8 |
| 68 | Ding et al. 2015 | 1 | 1 | 1 | 2 | 1 | 1 | 1 | 1 | 2 | 1 | 2 | 8 |
| 69 | Xu et al. 2014 | 1 | 1 | 1 | 1 | 1 | 1 | 1 | 1 | 2 | 2 | 3 | 8 |
| 70 | Zanetti et al. 2006 | 1 | 1 | 1 | 1 | 2 | 1 | 1 | 1 | 2 | 2 | 3 | 7 |
| 71 | Pioggiosi et al. 2006 | 1 | 1 | 1 | 2 | 1 | 1 | 1 | 1 | 2 | 2 | 2 | 7 |
| 72 | Manly et al. 2005 | 1 | 1 | 2 | 2 | 1 | 1 | 1 | 1 | 2 | 2 | 3 | 6 |
| 73 | Purser et al. 2005 | 1 | 1 | 1 | 1 | 1 | 1 | 2 | 2 | 2 | 2 | 3 | 6 |
| 74 | Kim et al. 2007 | 1 | 1 | 1 | 1 | 1 | 1 | 1 | 1 | 2 | 2 | 2 | 8 |
| 75 | Jungwirth et al. 2005 | 1 | 1 | 1 | 1 | 1 | 1 | 1 | 2 | 2 | 2 | 3 | 7 |
| 76 | Das et al. 2007 | 1 | 1 | 1 | 1 | 1 | 1 | 1 | 1 | 2 | 2 | 3 | 8 |
| 77 | Tognoni et al. 2005 | 1 | 1 | 1 | 1 | 1 | 1 | 1 | 1 | 2 | 2 | 3 | 8 |
| 78 | Boeve et al. 2003 | 1 | 1 | 1 | 1 | 1 | 1 | 1 | 1 | 2 | 2 | 3 | 8 |
| 79 | Ganguli et al. 2004 | 1 | 1 | 1 | 1 | 1 | 1 | 1 | 2 | 2 | 2 | 3 | 7 |
| 80 | Ravaglia et al. 2008 | 1 | 1 | 1 | 1 | 1 | 1 | 1 | 1 | 2 | 2 | 3 | 8 |
| 81 | Xie et al. 2003 | 1 | 2 | 1 | 3 | 3 | 1 | 2 | 1 | 2 | 2 | 2 | 4 |
| 82 | Yu et al. 2003 | 1 | 2 | 1 | 3 | 1 | 1 | 2 | 1 | 2 | 1 | 2 | 6 |
| 83 | Wu et al. 2005 | 1 | 1 | 2 | 3 | 1 | 1 | 2 | 2 | 2 | 2 | 2 | 4 |
| 84 | Yang et al. 2008 | 1 | 2 | 2 | 2 | 2 | 1 | 2 | 1 | 2 | 2 | 2 | 4 |
| 85 | Liu et al. 2007 | 1 | 2 | 2 | 1 | 1 | 1 | 2 | 1 | 2 | 1 | 2 | 6 |
| 86 | Wada-isoe et al. 2012 | 1 | 3 | 1 | 1 | 1 | 1 | 1 | 3 | 3 | 1 | 3 | 7 |
| 87 | Vlachos et al. 2020 | 1 | 3 | 3 | 3 | 1 | 1 | 2 | 1 | 2 | 2 | 2 | 4 |
| 88 | Bickel et al. 2006 | 1 | 2 | 2 | 1 | 1 | 1 | 1 | 1 | 1 | 2 | 1 | 8 |
| 89 | Busse et al. 2003 | 1 | 1 | 1 | 3 | 1 | 1 | 2 | 1 | 2 | 3 | 2 | 6 |
| 90 | Rahman et al. 2009 | 1 | 2 | 2 | 3 | 3 | 1 | 1 | 2 | 1 | 1 | 3 | 5 |
| 91 | Yu et al. 2003 | 1 | 2 | 2 | 1 | 3 | 1 | 1 | 1 | 1 | 1 | 3 | 7 |
| 92 | Assaf et al. 2021 | 1 | 1 | 1 | 1 | 1 | 1 | 1 | 1 | 2 | 2 | 3 | 8 |
| 93 | Eramudugolla et al. 2022 | 1 | 1 | 2 | 1 | 1 | 1 | 1 | 1 | 1 | 2 | 3 | 8 |
| 94 | Hussenoeder et al. 2020 | 1 | 1 | 1 | 1 | 1 | 1 | 1 | 1 | 2 | 2 | 3 | 8 |
| 95 | Mooldijk et al. 2022 | 1 | 1 | 1 | 1 | 1 | 1 | 1 | 1 | 2 | 2 | 3 | 8 |
| 96 | Nakahata et al. 2021 | 1 | 1 | 1 | 2 | 1 | 1 | 1 | 1 | 2 | 2 | 3 | 7 |
| 97 | Samson et al. 2022 | 1 | 1 | 1 | 1 | 1 | 1 | 1 | 1 | 2 | 2 | 3 | 8 |
| 98 | Lee et al. 2022 | 1 | 2 | 1 | 1 | 1 | 1 | 2 | 1 | 2 | 1 | 3 | 7 |
| 99 | Smith et al. 2022 | 1 | 2 | 2 | 1 | 3 | 1 | 1 | 1 | 1 | 1 | 2 | 7 |
| 100 | Xu et al. 2021 | 1 | 2 | 1 | 1 | 1 | 1 | 2 | 1 | 2 | 1 | 3 | 7 |
| 101 | Yamane et al. 2022 | 1 | 2 | 2 | 3 | 1 | 1 | 2 | 1 | 2 | 2 | 3 | 4 |
| 102 | Yang et al. 2021 | 1 | 2 | 1 | 1 | 1 | 1 | 2 | 1 | 2 | 1 | 3 | 7 |
| 103 | Yu et al. 2022 | 1 | 1 | 1 | 1 | 2 | 1 | 2 | 1 | 2 | 1 | 1 | 8 |
| 104 | Tang al.2007 | 1 | 1 | 1 | 1 | 1 | 1 | 2 | 1 | 2 | 2 | 3 | 7 |
| 105 | Gjøra et al, 2021 | 1 | 1 | 1 | 1 | 1 | 1 | 1 | 1 | 3 | 1 | 3 | 9 |
| 106 | Ramlall et al. 2013 | 1 | 1 | 2 | 1 | 3 | 1 | 1 | 3 | 2 | 1 | 2 | 6 |
| 107 | Yang et al. 2019 | 1 | 1 | 1 | 1 | 1 | 1 | 1 | 1 | 1 | 1 | 3 | 10 |
| 108 | Amoo et al. 2020 | 1 | 1 | 2 | 3 | 3 | 1 | 1 | 2 | 3 | 1 | 3 | 5 |
| 109 | Bae et al. 2017 | 1 | 1 | 2 | 1 | 3 | 1 | 1 | 3 | 3 | 1 | 2 | 6 |
| 110 | Fernández-Blázquez et al. 2021 | 1 | 1 | 1 | 1 | 3 | 1 | 1 | 3 | 1 | 1 | 3 | 8 |
| 111 | Ganguli et al. 2010 | 1 | 1 | 2 | 1 | 2 | 1 | 1 | 2 | 3 | 1 | 3 | 6 |
| 112 | González et al. 2019 | 1 | 1 | 2 | 1 | 1 | 1 | 1 | 2 | 3 | 1 | 1 | 8 |
| 113 | Guaita et al. 2015 | 1 | 1 | 1 | 1 | 1 | 1 | 1 | 3 | 2 | 1 | 3 | 8 |
| 114 | Heywood et al. 2017 | 1 | 1 | 1 | 1 | 1 | 1 | 1 | 3 | 2 | 1 | 1 | 9 |
| 115 | Kivipelto et al. 2001 | 1 | 1 | 2 | 1 | 3 | 1 | 1 | 2 | 3 | 1 | 3 | 6 |
| 116 | Lara et al. 2016 | 1 | 1 | 1 | 2 | 3 | 1 | 1 | 3 | 3 | 1 | 2 | 6 |
| 117 | Chong et al. 2019 | 1 | 1 | 1 | 3 | 2 | 1 | 1 | 2 | 3 | 1 | 3 | 6 |
| 118 | Das et al. 2007 | 1 | 1 | 1 | 3 | 3 | 1 | 1 | 3 | 1 | 1 | 3 | 7 |
| 119 | Juarez- Cedillo et al. 2012 | 1 | 1 | 1 | 1 | 3 | 1 | 1 | 3 | 2 | 1 | 2 | 7 |
| 120 | Ding et al. 2015 | 1 | 1 | 3 | 1 | 1 | 1 | 1 | 1 | 1 | 1 | 3 | 9 |
| 121 | Jia et al. 2014 | 1 | 1 | 1 | 1 | 1 | 1 | 1 | 2 | 3 | 1 | 2 | 8 |
| 122 | Jia et al. 2020 | 1 | 1 | 1 | 1 | 1 | 1 | 1 | 1 | 1 | 1 | 1 | 11 |
| 123 | Anstey et al. 2013 | 1 | 1 | 1 | 3 | 3 | 1 | 1 | 2 | 1 | 1 | 1 | 8 |
| 124 | Dimitrov et al. 2012 | 1 | 1 | 1 | 3 | 3 | 1 | 1 | 2 | 2 | 1 | 2 | 6 |
| 125 | Gavrila et al. 2009 | 1 | 1 | 1 | 3 | 3 | 1 | 1 | 2 | 2 | 1 | 2 | 6 |
| 126 | Han et al. 2017 | 1 | 1 | 1 | 1 | 3 | 1 | 1 | 2 | 3 | 1 | 3 | 7 |
| 127 | Hänninen et al. 2002 | 1 | 1 | 1 | 3 | 3 | 1 | 1 | 3 | 2 | 1 | 2 | 6 |
| 128 | Juncos-Rabadán et al. 2012 | 1 | 1 | 1 | 3 | 3 | 1 | 3 | 2 | 3 | 1 | 3 | 5 |
| 129 | Kim et al. 2011 | 1 | 1 | 2 | 2 | 3 | 1 | 1 | 3 | 3 | 1 | 3 | 5 |
| 130 | Limongi et al. 2017 | 1 | 1 | 1 | 1 | 2 | 1 | 1 | 1 | 1 | 1 | 2 | 9 |
| 131 | Liu et al. 2022 | 1 | 1 | 1 | 1 | 1 | 1 | 1 | 2 | 3 | 1 | 3 | 8 |
| 132 | Lopez-Anton et al. 2015 | 1 | 1 | 1 | 3 | 3 | 1 | 1 | 2 | 3 | 1 | 2 | 6 |
| 133 | Luck et al. 2007 | 1 | 1 | 1 | 1 | 1 | 1 | 1 | 2 | 1 | 1 | 3 | 9 |
| 134 | Mohan et al. 2019 | 1 | 1 | 1 | 1 | 3 | 1 | 1 | 2 | 1 | 1 | 2 | 8 |
| 135 | Mooi et al. 2016 | 1 | 1 | 1 | 3 | 3 | 1 | 1 | 1 | 1 | 1 | 2 | 8 |
| 136 | Moretti et al. 2013 | 1 | 1 | 1 | 1 | 3 | 1 | 1 | 2 | 1 | 1 | 1 | 9 |
| 137 | Noguchi-Shinohara et al. 2013 | 1 | 1 | 1 | 3 | 3 | 1 | 1 | 2 | 1 | 1 | 3 | 7 |
| 138 | Peltz et al. 2012 | 1 | 1 | 1 | 3 | 3 | 1 | 3 | 2 | 2 | 1 | 3 | 5 |
| 139 | Robertson et al. 2019 | 1 | 1 | 1 | 3 | 3 | 1 | 1 | 2 | 2 | 1 | 2 | 6 |
| 140 | Sasaki et al. 2009 | 1 | 1 | 1 | 3 | 2 | 1 | 1 | 2 | 3 | 2 | 2 | 5 |
| 141 | Shahnawaz et al. 2013 | 1 | 1 | 3 | 3 | 3 | 1 | 1 | 2 | 3 | 2 | 2 | 4 |
| 142 | Tsoy et al. 2019 | 1 | 1 | 1 | 3 | 2 | 1 | 1 | 3 | 1 | 1 | 2 | 7 |
| 143 | Radmila et al. 2019 | 1 | 1 | 1 | 3 | 3 | 1 | 1 | 3 | 1 | 1 | 1 | 8 |
| 144 | Vlachos et al. 2020 | 1 | 1 | 2 | 1 | 1 | 1 | 2 | 1 | 3 | 2 | 3 | 6 |
| 145 | Liu et al. 2022 | 1 | 1 | 1 | 1 | 3 | 1 | 1 | 3 | 3 | 1 | 2 | 7 |
| 146 | Su et al. 2014 | 1 | 1 | 3 | 3 | 3 | 1 | 1 | 1 | 3 | 1 | 3 | 6 |
| 147 | Mías et al. 2007 | 1 | 1 | 1 | 1 | 3 | 1 | 1 | 1 | 1 | 3 | 2 | 8 |
| 148 | Pedraza et al. 2017 | 1 | 1 | 1 | 2 | 3 | 1 | 1 | 1 | 3 | 1 | 1 | 8 |
| 149 | Sánchez et al. 2019 | 1 | 1 | 3 | 1 | 3 | 1 | 1 | 1 | 1 | 3 | 3 | 7 |
| 150 | Monteagudo Torres et al. 2009 | 1 | 1 | 1 | 1 | 3 | 1 | 1 | 2 | 3 | 3 | 3 | 6 |
| 151 | Wesseling et al. 2013 | 1 | 1 | 1 | 1 | 3 | 1 | 1 | 3 | 3 | 1 | 3 | 7 |
| 152 | Li et al. 2020 | 1 | 1 | 1 | 1 | 3 | 1 | 1 | 1 | 2 | 1 | 1 | 9 |
| 153 | Rao et al. 2018 | 1 | 1 | 1 | 2 | 3 | 1 | 1 | 1 | 2 | 2 | 1 | 7 |
| 154 | Sun et al. 2014 | 1 | 1 | 1 | 1 | 2 | 1 | 2 | 2 | 2 | 2 | 2 | 5 |
| 155 | Xiao et al. 2016 | 1 | 1 | 1 | 1 | 3 | 1 | 1 | 1 | 1 | 1 | 3 | 9 |
| 156 | Liu et al. 2018 | 1 | 1 | 1 | 3 | 3 | 1 | 1 | 2 | 3 | 1 | 3 | 6 |
| 157 | Wu et al. 2017 | 1 | 1 | 1 | 1 | 1 | 1 | 2 | 1 | 2 | 1 | 3 | 8 |
| 158 | Chuang et al. 2021 | 1 | 1 | 1 | 3 | 3 | 1 | 1 | 2 | 1 | 1 | 3 | 7 |
| 159 | Janelidze et al. 2018 | 1 | 1 | 3 | 1 | 1 | 1 | 2 | 1 | 3 | 2 | 2 | 6 |
| 160 | Pilleron et al. 2015 | 1 | 1 | 1 | 1 | 1 | 1 | 2 | 1 | 3 | 1 | 2 | 8 |
| 161 | Vancampfort et al. 2017 | 1 | 1 | 1 | 1 | 1 | 1 | 1 | 1 | 3 | 1 | 2 | 9 |
| 162 | Koyanagi et al.2019 | 1 | 1 | 1 | 1 | 1 | 1 | 2 | 1 | 3 | 2 | 2 | 7 |
| 163 | Li et al. 2013 | 1 | 1 | 1 | 1 | 1 | 1 | 1 | 1 | 2 | 2 | 3 | 8 |
| 164 | Kang et al. 2016 | 1 | 1 | 1 | 1 | 3 | 1 | 3 | 2 | 3 | 1 | 2 | 6 |
| 165 | Huang et al. 2021 | 1 | 1 | 1 | 1 | 3 | 1 | 3 | 2 | 2 | 1 | 3 | 6 |
| 166 | Bai et al. 2021 | 1 | 1 | 1 | 2 | 3 | 1 | 1 | 1 | 3 | 2 | 2 | 6 |
| 167 | Lu et al. 2022 | 1 | 1 | 1 | 1 | 3 | 1 | 3 | 2 | 3 | 1 | 2 | 6 |
| 168 | Shi et al. 2019 | 1 | 1 | 1 | 1 | 2 | 1 | 3 | 2 | 2 | 1 | 3 | 6 |
| 169 | Liu et al. 2005 | 1 | 1 | 1 | 1 | 3 | 1 | 3 | 2 | 3 | 3 | 2 | 5 |
| 170 | Sun et al. 2013 | 1 | 1 | 1 | 1 | 3 | 1 | 3 | 1 | 2 | 1 | 3 | 7 |
| 171 | Hai et al. 2010 | 1 | 1 | 1 | 1 | 3 | 1 | 3 | 3 | 3 | 2 | 1 | 6 |
| 172 | Yuan et al. 2017 | 1 | 1 | 1 | 1 | 2 | 1 | 1 | 1 | 2 | 1 | 3 | 8 |
| 173 | Ji et al. 2017 | 1 | 2 | 2 | 1 | 3 | 1 | 1 | 2 | 2 | 2 | 3 | 4 |
| 174 | Wang et al. 2013 | 1 | 1 | 2 | 1 | 3 | 1 | 1 | 2 | 2 | 1 | 3 | 6 |
| 175 | Zhao et al. 2015 | 1 | 2 | 1 | 1 | 2 | 1 | 3 | 1 | 2 | 2 | 3 | 5 |
| 176 | Li et al. 2013 | 1 | 2 | 2 | 1 | 2 | 1 | 3 | 1 | 2 | 1 | 3 | 5 |
| 177 | Pan et al. 2020 | 1 | 1 | 1 | 1 | 2 | 1 | 3 | 2 | 3 | 1 | 3 | 6 |
| 178 | Yu et al. 2012 | 1 | 1 | 1 | 1 | 2 | 1 | 2 | 1 | 3 | 1 | 3 | 7 |
| 179 | Yu et al. 2002 | 1 | 1 | 1 | 1 | 3 | 1 | 2 | 2 | 3 | 1 | 3 | 6 |
| 180 | Cai et al. 2010 | 1 | 1 | 1 | 1 | 3 | 1 | 3 | 2 | 3 | 1 | 1 | 7 |
| 181 | Chen et al. 2009 | 1 | 1 | 2 | 1 | 3 | 1 | 3 | 2 | 2 | 1 | 3 | 5 |
| 182 | Zhang et al. 2013 | 1 | 1 | 1 | 1 | 3 | 1 | 3 | 2 | 2 | 2 | 1 | 6 |
| 183 | Sun et al. 2008 | 1 | 1 | 1 | 1 | 3 | 1 | 3 | 2 | 3 | 2 | 3 | 5 |
| 184 | Yu et al. 2004 | 1 | 1 | 2 | 1 | 3 | 1 | 3 | 2 | 2 | 2 | 2 | 4 |
| 185 | Zhang et al. 2008 | 1 | 1 | 1 | 1 | 3 | 1 | 3 | 2 | 3 | 1 | 2 | 6 |
| 186 | Jiang et al. 2019 | 1 | 1 | 1 | 1 | 1 | 1 | 1 | 3 | 2 | 1 | 3 | 8 |
| 187 | Hu et al. 2012 | 1 | 1 | 1 | 1 | 3 | 1 | 1 | 2 | 2 | 2 | 2 | 6 |
| 188 | Guo et al. 2013 | 1 | 1 | 1 | 1 | 3 | 1 | 3 | 2 | 3 | 2 | 3 | 5 |
| 189 | Li et al. 2015 | 1 | 1 | 2 | 1 | 3 | 1 | 1 | 3 | 2 | 2 | 2 | 5 |
| 190 | Fan et al. 2014 | 1 | 1 | 1 | 1 | 3 | 1 | 3 | 2 | 3 | 2 | 3 | 5 |
| 191 | Lv et al. 2016 | 1 | 1 | 2 | 1 | 3 | 1 | 3 | 2 | 1 | 1 | 2 | 6 |
| 192 | Zhang et al. 2021 | 1 | 1 | 1 | 1 | 1 | 1 | 3 | 2 | 3 | 1 | 3 | 7 |
| 193 | Yuan et al. 2013 | 1 | 1 | 2 | 1 | 3 | 1 | 1 | 2 | 3 | 1 | 2 | 6 |
| 194 | Fang et al. 2015 | 1 | 1 | 2 | 1 | 3 | 1 | 2 | 1 | 2 | 2 | 3 | 5 |
| 195 | Pan et al. 2021 | 1 | 1 | 1 | 1 | 3 | 1 | 3 | 2 | 3 | 3 | 2 | 5 |
| 196 | Tao et al. 2016 | 1 | 1 | 1 | 1 | 3 | 1 | 1 | 2 | 2 | 1 | 3 | 7 |
| 197 | Li et al. 2021 | 1 | 1 | 1 | 1 | 2 | 1 | 3 | 2 | 2 | 2 | 2 | 5 |
| 198 | Xu et al. 2001 | 1 | 1 | 2 | 1 | 2 | 1 | 3 | 1 | 3 | 2 | 3 | 5 |
| 199 | Zhou et al. 2020 | 1 | 1 | 1 | 3 | 2 | 1 | 3 | 3 | 2 | 2 | 2 | 4 |
| 200 | Qiu et al. 2016 | 1 | 1 | 2 | 3 | 2 | 1 | 2 | 3 | 2 | 2 | 2 | 4 |
| 201 | Xia et al. 2011 | 1 | 1 | 1 | 3 | 2 | 1 | 2 | 2 | 3 | 2 | 2 | 4 |
| 202 | Wang et al. 2015 | 1 | 1 | 1 | 3 | 3 | 1 | 3 | 3 | 2 | 2 | 3 | 4 |
| 203 | Zhang et al. 2020 | 1 | 1 | 1 | 3 | 2 | 1 | 2 | 3 | 2 | 2 | 2 | 4 |
| 204 | Gao et al. 2011 | 1 | 1 | 1 | 1 | 1 | 1 | 3 | 1 | 3 | 1 | 3 | 8 |
| 205 | Xue et al. 2010 | 1 | 1 | 1 | 1 | 3 | 1 | 2 | 1 | 2 | 3 | 2 | 6 |
| 206 | Zhou et al. 2010 | 1 | 2 | 2 | 1 | 1 | 1 | 3 | 1 | 2 | 2 | 2 | 5 |
| 207 | Liang et al. 2008 | 1 | 2 | 2 | 1 | 3 | 1 | 2 | 1 | 3 | 2 | 3 | 4 |
| 208 | He et al. 2013 | 1 | 2 | 1 | 1 | 1 | 1 | 3 | 3 | 2 | 2 | 2 | 5 |
| 209 | Zhang et al. 2014 | 1 | 2 | 1 | 1 | 3 | 1 | 3 | 2 | 3 | 1 | 2 | 5 |
| 210 | Sun et al. 2012 | 1 | 1 | 1 | 1 | 2 | 1 | 2 | 3 | 2 | 2 | 2 | 5 |
| 211 | Sun et al. 2019 | 1 | 1 | 1 | 1 | 3 | 1 | 3 | 1 | 3 | 2 | 3 | 6 |
| 212 | Xiong et al. 2013 | 1 | 1 | 2 | 1 | 2 | 1 | 2 | 3 | 2 | 3 | 2 | 4 |
| 213 | Zhao et al. 2015 | 1 | 1 | 2 | 1 | 3 | 1 | 3 | 3 | 3 | 1 | 3 | 5 |
| 214 | Sun et al. 2013 | 1 | 1 | 1 | 1 | 2 | 1 | 2 | 2 | 3 | 2 | 2 | 5 |
| 215 | Song et al. 2019 | 1 | 1 | 1 | 1 | 2 | 1 | 1 | 3 | 2 | 3 | 2 | 6 |
| 216 | Wu et al. 2017 | 1 | 1 | 2 | 1 | 3 | 1 | 3 | 1 | 3 | 1 | 1 | 7 |
| 217 | Yang et al. 2016 | 1 | 1 | 2 | 1 | 2 | 1 | 2 | 3 | 2 | 1 | 2 | 5 |
| 218 | Su et al. 2016 | 1 | 1 | 2 | 1 | 2 | 1 | 3 | 1 | 3 | 2 | 2 | 5 |
| 219 | Xiang et al. 2009 | 1 | 1 | 2 | 1 | 2 | 1 | 3 | 3 | 2 | 1 | 3 | 5 |
| 220 | Xu et al. 2010 | 1 | 1 | 1 | 1 | 2 | 1 | 2 | 1 | 3 | 2 | 2 | 6 |
| 221 | Ma et al. 2019 | 1 | 1 | 1 | 1 | 2 | 1 | 3 | 3 | 3 | 2 | 2 | 5 |
| 222 | An et al. 2020 | 1 | 3 | 1 | 1 | 1 | 1 | 2 | 1 | 2 | 2 | 3 | 6 |
| 223 | Yang et al. 2019 | 1 | 1 | 1 | 1 | 2 | 1 | 1 | 3 | 2 | 1 | 2 | 7 |
| 224 | Liu et al. 2022 | 1 | 1 | 1 | 1 | 2 | 1 | 1 | 1 | 3 | 2 | 2 | 7 |
| 225 | Wang et al. 2017 | 1 | 1 | 1 | 1 | 2 | 1 | 2 | 1 | 2 | 3 | 2 | 6 |
| 226 | Wang et al. 2017 | 1 | 1 | 1 | 1 | 2 | 1 | 3 | 3 | 2 | 1 | 2 | 6 |
| 227 | Liu et al. 2021 | 1 | 1 | 1 | 1 | 2 | 1 | 3 | 1 | 2 | 2 | 2 | 6 |
| 228 | Zhou et al. 2013 | 1 | 1 | 1 | 1 | 3 | 1 | 2 | 3 | 2 | 2 | 2 | 5 |
| 229 | Jia et al. 2020 | 1 | 1 | 2 | 1 | 2 | 1 | 2 | 1 | 3 | 2 | 2 | 5 |
| 230 | Song et al. 2011 | 1 | 1 | 2 | 1 | 3 | 1 | 2 | 3 | 2 | 2 | 2 | 4 |
| 231 | Xu et al. 2016 | 1 | 1 | 1 | 3 | 1 | 1 | 3 | 3 | 2 | 1 | 3 | 6 |
| 232 | Ma et al. 2017 | 1 | 1 | 2 | 1 | 2 | 1 | 2 | 3 | 2 | 2 | 1 | 5 |
| 233 | Zhang et al. 2014 | 1 | 3 | 1 | 1 | 1 | 1 | 2 | 1 | 2 | 2 | 2 | 6 |

Notes: 1 = Yes; 2 = No; 3 = Unclear; Agency for Healthcare Research and Quality (AHRQ) methodology checklist, which included 11 items: (I) Define the source of information; (II) List inclusion and exclusion criteria for exposed and unexposed subjects or provide a reference to previous publications that describe these criteria; (III) Indicate time period used for identifying patients; (IV) Indicate whether or not subjects were consecutive if not population-based; (V) Indicate if evaluators of subjective components of were masked to other aspects of the status of the participants; (VI) Describe any assessments undertaken for quality control purposes; (VII) Explain any patient exclusions from analysis; (VIII) Describe how confounding was assessed and/or controlled; (IX) If applicable, explain how missing data were handled in the analysis; (X) Summarize patient response rates and completeness of data collection; (XI) Clarify what follow-up, if any, was expected and the percentage of patients for which incomplete data or follow-up was obtained. The quality score ranges from 0 to 11, with 1 point for each item, and is separated into three levels: low (0-3), moderate (4-7), and high (8-11).
