# Supplementary material for: Evidence from a meta-analysis and systematic review reveals the global prevalence of mild cognitive impairment
Source: Front Aging Neurosci. 2023 Oct 27;15:1227112. doi: 10.3389/fnagi.2023.1227112 (PMC10641463; doi:10.3389/fnagi.2023.1227112)
Supplement: Supplementary file 3 [file Data_Sheet_3.docx]

**Supplementary File 3. All included studies in this systematic review and meta-analysis**

1. Björk, S., Lövheim, H., Lindkvist, M., Wimo, A., and Edvardsson, D. (2018). Thriving in relation to cognitive impairment and neuropsychiatric symptoms in Swedish nursing home residents. *International journal of geriatric psychiatry*, 33(1), e49-e57. doi: 10.1002/gps.4714
2. Tiwari, S. C., Srivastava, G., Tripathi, R. K., Pandey, N. M., Agarwal, G. G., Pandey, S., et al. (2013). Prevalence of psychiatric morbidity amongst the community dwelling rural older adults in northern India. *The Indian journal of medical researc*h, 138(4), 504-514.
3. Rao, D., Luo, X., Tang, M., Shen, Y., Huang, R., Yu, J., et al. (2018). Prevalence of mild cognitive impairment and its subtypes in community-dwelling residents aged 65 years or older in Guangzhou, China. *Archives of Gerontology and Geriatrics*, 75, 70-75. doi: 10.1016/j.archger.2017.11.003
4. Vancampfort, D., Stubbs, B., Lara, E., Vandenbulcke, M., Swinnen, N., and Koyanagi, A. (2017). Mild cognitive impairment and physical activity in the general population: Findings from six low-and middle-income countries. Experimental gerontology, 100, 100-105. doi: 10.1016/j.exger.2017.10.028
5. Lu, H., Wang, X. D., Shi, Z., Yue, W., Zhang, Y., Liu, S., et al. (2019). Comparative analysis of cognitive impairment prevalence and its etiological subtypes in a rural area of northern China between 2010 and 2015. *Scientific Reports*, 9(1), 1-7. doi: 10.1038/s41598-018-37286-z
6. Su, X. N., Hua, Q. Z., Zhang, L., Li, N. N., Cheng, J. H., and Zhang, L. P. (2013). Influencing factors of mild cognitive impairment of seniors in communities of Xi’an. *J Nurs*, 11, 10-13. doi: 10.16460/j.issn1008-9969.2013.11.028
7. Zhang, J., Jiang, H., Wang, F., and Gao, L. (2013). Investigation and analysis on mild cognitive impairment among the elderly in the communities of Taicang city. *Practical Geriatrics*, 27(10), 859-862. doi: 10.3969/i.issn.10039198.2013.10.021
8. Zhang, W., Li, C., Tian, F., Wang, Y., Liang, Y., Liu, X. (2015). Value of the Montreal Cognitive Assessment Scale (Beijing version) for screening mild cognitive impairment in rural older adults. *Chinese Journal Of Gerontology*, 35(14), 4016-4018. doi: 10. 3969 /j. issn.1005-9202.2015.14.112
9. Li, Z., Huang, Y., Liu, J., Yang, H., Liu, S., Wang, Y. (2013). Risk factors to inducing mild cognitive impairment of the aged in Nanyan area. *J am geriatr soc*, 29(04), 406-408. doi: 10.13329/j.cnki.zyyjk.2013.04.047
10. Guo, G. Y., Tan, G. Y., Zhang, S. F., Yu, X. D., and Li, C. L. (2013). Status analysis of mild cognitive im pairment of the elderly in Chengde City. *China Medical Herald*, 10(6), 110-1.
11. Yin, L., Cui, Z., Yang, H., Guo, Z., Shao, C., Lu, P. (2012). Investigation on mild cognitive impairment. *Clin Focus*, 27(23), 2032-2035.
12. Pan, H. Y., Wang, J. Q., Wu, M., and Chen, J. J. (2012). Study on prevalence rate and quality of life of elderly patients with mild cognitive impairment in community. *Nurs J Chin PLA*, 29(1B), 6-9.
13. Xia, Y., Chen, B., Su, X., Ni, Y., Jiang, C., Li, X., et al. (2006). Cognitive functioning in community-dwelled aged:a comparison of baseline and 4-year follow up. *Chin J Psychiatry*, 2006(01), 29-32.
14. Yang Y, H., Cheng, G., Rong, S., Zhu, H., Chen, D., Yang, Bei., et al. (2017). A cross-sectional study on mild cognitive impairment among the elderly in communities of Huangshi City. *Chin J Dis Control Prev*, 21(8), 767-771. doi: 10. 16462/j. enki. zhjbkz. 2017. 08. 004
15. Zhang, Y., Wang, Z. L., Xu, H., Wang, H., Yang, Z. J., Peng, L., et al. (2018). Investigation of prevalence and factors influencing mild cognitive impairment in elderly community residents in Shanghai. *Chin Gen Pract*, 21(24), 2909. doi: 10.12114/j.issn. 1007-9572.2018.00.009
16. Jiang, H., Wang, X. M., Huang, K. Y., Zuo, Y. K., Wu, X. M., Gao, Y. F., et al. (2019). Study on prevalence of and influencing factors of mild cognitive impairment among elderly people in communities of Nanning. *Chin J Dis Cont Prev*, 313-317. doi: 10.16462/j.cnki.zhibkz.2019.03.014
17. Dai, F., Kong, F., Cheng, J., and Wang, C. (2019). Prevalence rate and influencing factors of mild cognitive impairment in the elderly in Qingdao community. *J Psychiatry*, 32(03), 195-199. doi: 10.3969/i.issn.2095-9346.2019.03.008
18. Liu, J. L., Yu, D. H., Lu, Y., and Wang, H. (2019). Screening of mild cognitive impairment among the elderly in urban and rural areas of Shanghai and analysis on its related factors. *China Med Herald,* 16(24), 62-6.
19. Yuan, F., Wang, L., Wang, Z. (2019). The Association between Food Patterns and Mild Cognitive Impairment in Community Residents aged 55 Years and Older. *World Latest Med Inf*, 19(26), 25-27. doi: 10.19613/j.cnki.1671-3141.2019.26.012
20. Yuan, M., Wei, X., Chen, J., Yang, X., Han, Y., Fang Y. (2021). Prevalence and influencing factors of mild cognitive impairment among elderly residents in urban and rural Xiamen. *Chin J Public Health*, 19(26), 25-27. doi: 10.19613/j.cnki.1671-3141.2019.26.012
21. Luo, J., Wu, B., Zhao, Q., Guo, Q., Meng, H., Yu, L., et al. (2015). Association between tooth loss and cognitive function among 3063 Chinese older adults: a community-based study. *PloS one*, 10(3), e0120986. doi: 10.1371/journal.pone.0120986
22. Xu, S., Xie, B., Song, M., Yu, L., Wang, L., An, C., et al. (2014). High prevalence of mild cognitive impairment in the elderly: a community-based study in four cities of the Hebei Province, China. *Neuroepidemiology*, 42(2), 123-130. doi: 10.1159/000357374
23. Tang, Z., Zhang, X., Wu, X., Liu, H., Diao, L., Guan, S., et al. (2007). Prevalence of the mild cognitive impairment among elderly in Beijing. *Chinese Mental Health Journal*, 2007(02), 116-118.
24. Guan, S., Tang, Z., Wu, X., Diao, L., Liu, H., Sun, F., et al. (2008). Investigation of the incidence of mild cognitive impairment and its risk factors in an elderly population sample in Beijing area. *Chin J Cerebrovasc Dis*, 2008(09), 395-398.
25. Huang, R. Y., Tang, M. N., Ma, C., Guo, Y. B., Han, H. Y., and Huang, J. M. (2008). The prevalence of mild cognitive impairment of residents aged 60 years and over in the urban and rural areas in Guangzhou. *Chin J Nerv Ment Dis*, 34(9), 533-7.
26. Ren, C. F., Yuan, Y. F., Zhang, Y., Wang, J. L., Lu, H. L., and Wan, A. L. (2013). Analysis on prevalence and influencing factors of mild cognitive impairment among the elderly in Jiangxi Communitie. *Modem Prev Med*, 40(21), 3969-73.
27. Zhou, D., Xu, Y., Cheng, Z., Hu, Z., and Chen, Y. (2011). Prevalence of mild cognitive impairment among the elderly. *Chin J Public Health*, 27(11), 1375-1377.
28. Chen, Y., Liu, H., Feng, H., Jiang, H., and Zhuang, X. (2015). Investigation on cognitive impairment of the elderly in downtown center of Shanghai. Chinese Journal Of Rehabilitation Medicine, 30(04), 391-392.
29. Pan, Z. D., Zhou, R. S., Wang, T., and Xian, S. F. (2012). Prevalence of mild cognitive impairment among the elderly in community. *Geriatr Health Care*, 18(3), 154-6. doi: 10.3969/j.issn.1008-8296.2012-03-09
30. Song, X. Z., Chen, J. H., and He, L. P. (2012). Investigation on correlation between prevalence of the mild cognitive impairment and eating habit in elderly in the communities of Shunde city. *IMHGN*, 18(12), 1715-8. doi: 10.3760/cma.j.issn.1007-1245.2012.12.009
31. Zhu, X. Q., Zhou, X. H., Kumusi, B., Yue, Y. H., Zhao, R. J., and Xin, S. F. (2009). Study of prevalence of the mild cognitive impairment among elderly in the communities of Urumqi city. *J Xinjiang Med Univ*, 32(5), 578-84.
32. Wu, B., Zhang, L. Y., Su, Y. L., Dang, Y. H., and Hou, J. X. (2012). Investigation on mild cognitive impairment among elderly in urban community of Xi’an. *Zhongguo Kang Fu Li Lun Yu Shi Jian*, 18(7), 605-607. doi: 10.3969/j.issn.1006-9771.2012.07.002
33. Liao, B., Gao, R., Xiong, L. H., Yi, G. P., Li, Y., and Wan, S. L. (2012). The early evaluation and intervention strategies of mild cognitive impairment in Yichun area. *J Yichun Coll*, 24, 77-79.
34. Zhang, X. Q., and Hu, Z. (2014). Prevalence and factors associated with mild cognitive impairment among the elderly in Changsha communities. *Chin Gen Pract*, 17(9), 1031-5. doi: 10.3969/j.issn.1007-9572.2014.09.014
35. Afgin, A. E., Massarwa, M., Schechtman, E., Israeli-Korn, S. D., Strugatsky, R., Abuful, A., Farrer, L. A., Friedland, R. P., & Inzelberg, R. (2012). High prevalence of mild cognitive impairment and Alzheimer's disease in arabic villages in northern Israel: impact of gender and education. Journal of Alzheimer's disease : JAD, 29(2), 431–439. https://doi.org/10.3233/JAD-2011-111667
36. Artero, S., Ancelin, M. L., Portet, F., Dupuy, A., Berr, C., Dartigues, J. F., et al. (2008). Risk profiles for mild cognitive impairment and progression to dementia are gender specific. *Journal of Neurology, Neurosurgery & Psychiatry*, 79(9), 979-984. doi: 10.1136/jnnp.2007.136903
37. Lee, K. S., Cheong, H. K., Oh, B. H., Na, D. L., and Hong, C. H. (2009). Working criteria of mild cognitive impairment in community: findings from Gwangju Dementia and Mild Cognitive Impairment Study (GDEMCIS). *Asia‐Pacific Psychiatry*, 1(1), 15-22. doi: 10.1111/j.1758-5872.2009.00004.x
38. Ogunniyi, A., Adebiyi, A. O., Adediran, A. B., Olakehinde, O. O., & Siwoku, A. A. (2016). Prevalence estimates of major neurocognitive disorders in a rural Nigerian community. *Brain and Behavior*, 6(7), e00481. doi: 10.1002/brb3.481
39. Petersen, R. C., Roberts, R. O., Knopman, D. S., Geda, Y. E., Cha, R. H., Pankratz, V. S., et al. (2010). Prevalence of mild cognitive impairment is higher in men: The Mayo Clinic Study of Aging. *Neurology*, 75(10), 889-897. doi: 10.1212/WNL.0b013e3181f11d85
40. Pilleron, S., Jésus, P., Desport, J. C., Mbelesso, P., Ndamba-Bandzouzi, B., Clément, J. P., et al. (2015). Association between mild cognitive impairment and dementia and undernutrition among elderly people in Central Africa: some results from the EPIDEMCA (Epidemiology of Dementia in Central Africa) programme. *British Journal of Nutrition*, 114(2), 306-315. doi: 10.1017/S0007114515001749
41. Richard, E., Reitz, C., Honig, L. H., Schupf, N., Tang, M. X., Manly, J. J., et al. (2013). Late-life depression, mild cognitive impairment, and dementia. *JAMA Neurology*, 70(3), 383-389. doi: 10.1001/jamaneurol.2013.603
42. Kumar, R., Dear, K. B., Christensen, H., Ilschner, S., Jorm, A. F., Meslin, C., et al. (2005). Prevalence of mild cognitive impairment in 60-to 64-year-old community-dwelling individuals: The Personality and Total Health through Life 60+ Study. *Dementia And Geriatric Cognitive Disorders*, 19(2-3), 67-74. doi: 10.1159/000082351
43. Lee, S. B., Kim, K. W., Youn, J. C., Park, J. H., Lee, J. J., Kim, M. H., et al. (2009). Prevalence of mild cognitive impairment and its subtypes are influenced by the application of diagnostic criteria: results from the Korean Longitudinal Study on Health and Aging (KLoSHA). *Dementia And Geriatric Cognitive Disorders*, 28(1), 23-29. doi: 10.1159/000228841
44. Lee, L. K., Shahar, S., Chin, A. V., Yusoff, N. A. M., Rajab, N., and Aziz, S. A. (2012). Prevalence of gender disparities and predictors affecting the occurrence of mild cognitive impairment (MCI). *Archives of gerontology and geriatrics*, 54(1), 185-191. doi: 10.1016/j.archger.2011.03.015
45. Purser, J. L., Fillenbaum, G. G., Pieper, C. F., and Wallace, R. B. (2005). Mild cognitive impairment and 10‐year trajectories of disability in the Iowa established populations for epidemiologic studies of the elderly cohort. *Journal of the American Geriatrics Society*, 53(11), 1966-1972. doi: 10.1111/j.1532-5415.2005.53566.x
46. De Jager, C. A., and Budge, M. M. (2005). Stability and predictability of the classification of mild cognitive impairment as assessed by episodic memory test performance over time. *Neurocase*, 11(1), 72-79. doi: 10.1080/13554790490896820
47. Khedr, E., Fawi, G., Abbas, M. A. A., Mohammed, T. A., El-Fetoh, N. A., Al Attar, G., et al. (2015). Prevalence of mild cognitive impairment and dementia among the elderly population of Qena Governorate, Upper Egypt: a community-based study. *Journal of Alzheimer's Disease*, 45(1), 117-126. doi: 10.3233/JAD-142655
48. Yu, J., Lam, C. L., and Lee, T. M. (2016). Perceived loneliness among older adults with mild cognitive impairment. *International Psychogeriatrics*, 28(10), 1681-1685. doi: 10.1017/S1041610216000430
49. Ma, F., Wu, T., Zhao, J., Ji, L., Song, A., Zhang, M., and Huang, G. (2016). Prevalence of mild cognitive impairment and its subtypes among Chinese older adults: role of vascular risk factors. *Dementia and Geriatric Cognitive Disorders*, 41(5-6), 261-272. doi: 10.1159/000446507
50. Wang, Y., Song, M., Yu, L., Wang, L., An, C., Xun, S., et al. (2015). Mild cognitive impairment: vascular risk factors in community elderly in four cities of Hebei Province, China. *PloS one*, 10(5), e0124566. doi: 10.1371/journal.pone.0124566
51. Jia, J., Zhou, A., Wei, C., Jia, X., Wang, F., Li, F., et al. (2014). The prevalence of mild cognitive impairment and its etiological subtypes in elderly Chinese. *Alzheimer's & Dementia*, 10(4), 439-447. doi: 10.1016/j.jalz.2013.09.008
52. Hu, R., Zhao, S. G., Wang, D. S., Wen, S. R., Niu, G. M., Wang, Z. G., et al. (2012). A prevalence study on mild cognitive impairment among the elderly populations of Mongolian and Han nationalities in a pastoral area of Inner Mongolia. *Chin J Epidemiol*, 33(4), 364-367. doi: 10.3760/cma.j.issn.0254-6450.2012.04.004
53. Qiu, C. J., Tang, M. N., Zhang, W., Han, H. Y., Dai, J., Lu, J., et al. (2003). The prevalence of mild cognitive impairment among residents aged 55 or over in Chengdu area. *Chin J Epidemiol*, 24(12), 1104-1107.
54. Lei, M., Huang, W., Yang, J., Yang, X., Deng, H., and Zhang, N. (2008). Prevalence of mild cognitive impairment among old people in urban and rural areas of Guizhou Province. *Chinese Mental Health Journal*, 2008(05), 387-391.
55. Lao, M. L., Zhang, H. Y., Luo, G., Yi, X. N., Huang, Y. D., and Wu, Z. H. (2011). Prevalence of mild cognitive impairment amony 55-years old or over individuals in Hainan Island. *Hainan Med J*, 22(14), 112-4.
56. Yang, L., and Qin, Q. B. (2011). Study on prevalence and risk factors of mild cognitive impairment among retired cadres. *Chin J Nervous Mental Dis*, 37(8), 473-6.
57. Yin, S. Q., Nie, H. W., and Xu, Y. (2011). The prevalence and risk factors of mild cognitive impairment among the aged in Huzhou. *Chin Gen Pract*, 14, 4145-4147.
58. Tong, J. F., Guo, S. Y., Tao, X. J., Guo, J. X., Tian, Y., and Xia, B. J. (2013). The elderly patients with mild cognitive impairment in the community of Tangshan. *Chin J Helth Psychol*, 21, 1642-1644. doi: 10.13342/j.enki.cjhp.2013.11.058
59. Xiong, Y., Miao, R. J., Wang, Q. Q., Zhou, L. J., Gao, L., and Ma, F. (2013). Prevalence and influencing factors of MCI among community elderly in Tianjin city. *Chin J Public Health*, 29(1), 1-4. doi: 10.11847/zgggws2013-29-01-01
60. Zhang, J., Jiang, H., Wang, F., and Gao, L. (2013). Investigation and analysis on mild cognitive impairment among the elderly in the communities of Taicang city. *Practical Geriatrics*, 27(10), 859-862. doi: 10.3969/j. issn. 1003-9198.2013.10.021
61. Gu, B., Yan, G., and Zhou, S. (2014). The epidemiology of mild cognitive impairment and intervention effect of folic acid in Hefei. *Practical geriatrics*, 17(05), 475-477. doi: 10.3969/J. issn. 1672-6790.2014.05.010
62. Qin, H. Y., Chen, D. H., and Qu, Z. W. (2014). Investigation of mild cognitive impairment and its risk factors among 55 years old and above residents in Shanghai. *J Clin Psychiatry*, 24(3), 155-158. doi: CNKI:SUN:LCJS.0.2014-03-007
63. Sun, H. Y., Qu, Q. M., Liu, J., Zhang, J., Guo, X., and JIa, L. (2016). The epidemiological study of ethnic disparity in risk factors for mild cognitive impairment between Mongolia and Han population in baotou, Inner Mongolia. *J Apoplexy Nerv Dis*, 33, 454-456. doi: 10.19845/j.cnki.zfysjjbzz.2016.05.016
64. Zhou, D. M., Chen, Q., Sui, C. Y., and Yang, M. (2016). Investigation on the prevalence of mild cognitive impairment in the elderly in Changji city. *Chin Community Doct*, 32, 178-183. doi: 10.3969/j.issn.1007-614x.2016.5.114
65. Guo, M., Gao, L., Zhang, G., Li, Y., Xu, S., Wang, Z., et al (2012). Prevalence of dementia and mild cognitive impairment in the elderly living in nursing and veteran care homes in Xi'an, China. *Journal of the neurological sciences*, 312(1-2), 39-44. doi: 10.1016/j.jns.2011.08.026
66. Jia, J., Zhou, A., Wei, C., Jia, X., Wang, F., Li, F., et al. (2014). The prevalence of mild cognitive impairment and its etiological subtypes in elderly Chinese. *Alzheimer's & Dementia*, 10(4), 439-447. doi: 10.1016/j.jalz.2013.09.008
67. Li, X., Ma, C., Zhang, J., Liang, Y., Chen, Y., Chen, K., et al. (2013). Prevalence of and potential risk factors for mild cognitive impairment in community‐dwelling residents of Beijing. *Journal of the American Geriatrics Society*, 61(12), 2111-2119. doi: 10.1111/jgs.12552
68. Ding, D., Zhao, Q., Guo, Q., Meng, H., Wang, B., Luo, J., et al. (2015). Prevalence of mild cognitive impairment in an urban community in China: a cross-sectional analysis of the Shanghai Aging Study. *Alzheimer's & Dementia*, 11(3), 300-309. doi: 10.1016/j.jalz.2013.11.002
69. Xu, S., Xie, B., Song, M., Yu, L., Wang, L., An, C., and Wang, X. (2014). High prevalence of mild cognitive impairment in the elderly: a community-based study in four cities of the Hebei Province, China. *Neuroepidemiology*, 42(2), 123-130. doi: 10.1159/000357374
70. Zanetti, M., Ballabio, C., Abbate, C., Cutaia, C., Vergani, C., and Bergamaschini, L. (2006). Mild cognitive impairment subtypes and vascular dementia in community‐dwelling elderly people: a 3‐year follow‐up study. *Journal of the American Geriatrics Society*, 54(4), 580-586. doi: 10.1111/j.1532-5415.2006.00658.x
71. Pioggiosi, P. P., Berardi, D., Ferrari, B., Quartesan, R., and De Ronchi, D. (2006). Occurrence of cognitive impairment after age 90: MCI and other broadly used concepts. *Brain research bulletin*, 68(4), 227-232. doi: 10.1016/j.brainresbull.2005.06.039
72. Manly, J. J., Bell-McGinty, S., Tang, M. X., Schupf, N., Stern, Y., and Mayeux, R. (2005). Implementing diagnostic criteria and estimating frequency of mild cognitive impairment in an urban community. *Archives of neurology*, 62(11), 1739-1746. doi: 10.1001/archneur.62.11.1739
73. Purser, J. L., Fillenbaum, G. G., Pieper, C. F., and Wallace, R. B. (2005). Mild cognitive impairment and 10‐year trajectories of disability in the Iowa established populations for epidemiologic studies of the elderly cohort. *Journal of the American Geriatrics Society*, 53(11), 1966-1972. doi: 10.1111/j.1532-5415.2005.53566.x
74. Kim, J., Park, M. H., Kim, E., Han, C., Jo, S. A., and Jo, I. (2007). Plasma homocysteine is associated with the risk of mild cognitive impairment in an elderly Korean population. *The Journal of nutrition*, 137(9), 2093-2097. doi: 10.1093/jn/137.9.2093
75. Jungwirth, S., Weissgram, S., Zehetmayer, S., Tragl, K. H., and Fischer, P. (2005). VITA: subtypes of mild cognitive impairment in a community‐based cohort at the age of 75 years. *International Journal of Geriatric Psychiatry: A journal of the psychiatry of late life and allied sciences*, 20(5), 452-458. doi: 10.1002/gps.1311
76. Das, S. K., Bose, P., Biswas, A., Dutt, A., Banerjee, T. K., Hazra, A., et al. (2007). An epidemiologic study of mild cognitive impairment in Kolkata, India. *Neurology*, 68(23), 2019-2026. doi: 10.1212/01.wnl.0000264424.76759.e6
77. Tognoni, G., Ceravolo, R., Nucciarone, B., Bianchi, F., Dell'Agnello, G., Ghicopulos, I., et al. (2005). From mild cognitive impairment to dementia: a prevalence study in a district of Tuscany, Italy. Acta neurologica scandinavica, 112(2), 65-71. doi: 10.1111/j.1600-0404.2005.00444.x
78. Boeve, B., McCormick, J., Smith, G., Ferman, T., Rummans, T., Carpenter, T., et al. (2003). Mild cognitive impairment in the oldest old. *Neurology*, 60(3), 477-480. doi: 10.1212/WNL.60.3.477
79. Ganguli, M., Dodge, H. H., Shen, C., and DeKosky, S. T. (2004). Mild cognitive impairment, amnestic type: an epidemiologic study. Neurology, 63(1), 115-121. doi: 10.1212/01.WNL.0000132523.27540.81
80. Ravaglia, G., Forti, P., Montesi, F., Lucicesare, A., Pisacane, N., Rietti, E., et al. (2008). Mild cognitive impairment: epidemiology and dementia risk in an elderly Italian population. Journal of the American Geriatrics Society, 56(1), 51-58. doi: 10.1111/j.1532-5415.2007.01503.x
81. Xie, H., Wang, X., Ma, T., Wang, Z., and Wang, L. (2003). Mild cognitive impairment and its risk factors in people aged 75 years and older：a population-based preliminary study. *Chinese Journal Of Geriatric Heart Brain And Vessel Diseases*, 2003(05), 318-321. doi: 10.3969/j.issn.1009-0126.2003.05.009
82. YU, B., Wang, Y., Tian, J., Ouyang, L., LI, Q., Zhang, L., et al. (2003). Study of the prevalence of mild cognitive impairment in elderly veterans. *Chinese Journal of Geriatrics*, 2003(01), 39-41. doi: 10.3760/j:issn:0254-9026.2003.01.011
83. Wu, Y. G., Li, Z. H. B., Zhang, G. B., Zhang, L. G., and Xu, Z. Y. (2005). A preliminary study of the epidemiology of succeddful aging, usual aging and mild cognitive impairment in Bma elderly. *J Guangxi Med Univ*, 22, 366-367. doi: 10.16190/j.cnki.45-1211/r.2005.03.015
84. Yang, J. Y., Huang, W. Y., Yang, X., Wang, J. H., Lei, M. Y., and Zhang, N. (2008). Investigation on cognitive impairment among elders in urban community of Guiyang. *Chinese Journal of Public Health*, 24(10), 1167-1168. doi: 10.3321/j.issn:1001-0580.2008.10.012
85. Liu, J. L., Gao, H., Song, F., Li, J. L., and Shi, W. (2007). Analysis of risk factors of cognitive handicap and senile dementia in honorary retired and ordinary retired cadres from 51 cadre sanatoriurns of 11areas in Yellow River Valley. *J Clin Rehabilitative Tissue Eng Res*, 11, 5869-5871.
86. Wada-Isoe, K., Uemura, Y., Nakashita, S., Yamawaki, M., Tanaka, K., Yamamoto, M., et al. (2012). Prevalence of dementia and mild cognitive impairment in the rural island town of Ama-cho, Japan. *Dementia and geriatric cognitive disorders extra*, 2(1), 190-199. doi: 10.1159/000338244
87. Vlachos, G. S., Kosmidis, M. H., Yannakoulia, M., Dardiotis, E., Hadjigeorgiou, G., Sakka, P., et al. (2020). Prevalence of mild cognitive impairment in the elderly population in Greece: results from the HELIAD study. *Alzheimer Disease & Associated Disorders*, 34(2), 156-162. doi: 10.1097/WAD.0000000000000361
88. Bickel, H., Mösch, E., Seigerschmidt, E., Siemen, M., and Förstl, H. (2006). Prevalence and persistence of mild cognitive impairment among elderly patients in general hospitals. *Dementia and Geriatric Cognitive Disorders*, 21(4), 242-250. doi: 10.1159/000091397
89. Busse, A., Bischkopf, J., Riedel‐Heller, S. G., and Angermeyer, M. C. (2003). Mild cognitive impairment 1: Prevalence and predictive validity according to current approaches. *Acta Neurologica Scandinavica*, 108(2), 71-81. doi: 10.1034/j.1600-0404.2003.00118.x
90. Rahman, T. T. A., and El Gaafary, M. M. (2009). Montreal Cognitive Assessment Arabic version: reliability and validity prevalence of mild cognitive impairment among elderly attending geriatric clubs in Cairo. *Geriatrics & gerontology international*, 9(1), 54-61. doi: 10.1111/j.1447-0594.2008.00509.x
91. Yu, B., Wang, Y., Tian, J., Wang, C., Ouyang, J., Wang, L., et al. (2003). Prevalence of mild cognitive impairment: a population-based study in elderly veterans. *Chinese Journal Of Clinical Rehabilitation*, 7(3), 496-497.
92. Assaf, G., El Khoury, J., Jawhar, S., and Rahme, D. (2021). Mild Cognitive Impairment and modifiable risk factors among Lebanese older adults in primary care. *Asian Journal of Psychiatry*, 65, 102828. doi: 10.1016/j.ajp.2021.102828
93. Eramudugolla, R., Huynh, K., Zhou, S., Amos, J. G., and Anstey, K. J. (2022). Social cognition and social functioning in MCI and dementia in an epidemiological sample. *Journal of the International Neuropsychological Society*, 28(7), 661-672. doi: 10.1017/S1355617721000898
94. Hussenoeder, F. S., Conrad, I., Roehr, S., Fuchs, A., Pentzek, M., Bickel, H., et al. (2020). Mild cognitive impairment and quality of life in the oldest old: a closer look. *Quality of Life Research*, 29, 1675-1683. doi: 10.1007/s11136-020-02425-5
95. Mooldijk, S. S., Yaqub, A., Wolters, F. J., Licher, S., Koudstaal, P. J., Ikram, M. K., and Ikram, M. A. (2022). Life expectancy with and without dementia in persons with mild cognitive impairment in the community. *Journal of the American Geriatrics Society*, 70(2), 481-489. doi: 10.1111/jgs.17520
96. Nakahata, N., Nakamura, T., Kawarabayashi, T., Seino, Y., Ichii, S., Ikeda, Y., et al. (2021). Age-related cognitive decline and prevalence of mild cognitive impairment in the Iwaki health promotion project. *Journal of Alzheimer's Disease*, 84(3), 1233-1245. doi: 10.3233/JAD-210699
97. Samson, A. D., Shen, K., Grady, C. L., McIntosh, A. R., and Alzheimer's Disease Neuroimaging Initiative. (2022). Exploration of salient risk factors involved in mild cognitive impairment. *European Journal of Neuroscience*, 56(9), 5368-5383. doi: 10.1111/ejn.15665
98. Smith, L., Pizzol, D., López Sánchez, G. F., Kostev, K., Oh, H., Jacob, L., et al. (2022). Association between cooking fuels and mild cognitive impairment among older adults from six low-and middle-income countries. *Scientific Reports*, 12(1), 1-8. doi: 10.1038/s41598-022-17216-w
99. Smith, L., Shin, J. I., Oh, H., Carmichael, C., Jacob, L., Stefanac, S., et al. (2022). Body mass index and mild cognitive impairment among middle-aged and older adults from low-and middle-income countries. *Journal of Alzheimer's Disease, (Preprint)*, 1-11. doi: 10.3233/JAD-215345
100. Xu, L., Wei, L., Zhang, Y., Zhu, Z., Li, Y., and Chen, W. (2021). Application of Screening Scale for Mild Cognitive Impairment in screening mild cognitive impairment of the elderly in rural communities in Hangzhou, Zhejiang. *Chin J Contemp Neurol Neurosurg*, 21(12), 1057-1063.
101. Yamane, N., Ikeda, A., Tomooka, K., Saito, I., Maruyama, K., Eguchi, E., et al. (2022). Salivary Alpha-Amylase Activity and Mild Cognitive Impairment among Japanese Older Adults: The Toon Health Study. *The Journal of Prevention of Alzheimer's Disease*, 9(4), 752-757. doi: 10.14283/jpad.2022.51
102. Yang, J., Zhao, X., Sui, H., and Liu, X. (2021). High prevalence and low awareness of mild cognitive impairment in a suburban community in Shanghai. *Neurology India*, 69(6), 1693. doi: 10.4103/0028-3886.333524
103. Yu, K., Wild, K., Dowling, N. M., Kaye, J. A., Silbert, L. C., and Dodge, H. H. (2022). Emotional characteristics of socially isolated older adults with MCI using tablet administered NIH toolbox: I‐CONECT study. *Alzheimer's & Dementia: Diagnosis, Assessment & Disease Monitoring*, 14(1), e12372.
104. Tang, Z., Zhang, X., Wu, X., Liu, H., Diao, L., Guan, S., et al. (2007). Prevalence of the mild cognitive impairment among elderly in Beijing. *Chinese Mental Health Journal*, 2007(02), 116-118.
105. Gjøra, L., Strand, B. H., Bergh, S., Borza, T., Brækhus, A., Engedal, K., et al. (2021). Current and future prevalence estimates of mild cognitive impairment, dementia, and its subtypes in a population-based sample of people 70 years and older in Norway: the HUNT study. *Journal of Alzheimer's Disease*, 79(3), 1213-1226. doi: 10.3233/JAD-201275
106. Ramlall, S., Chipps, J., Pillay, B. J., and Bhigjee, A. I. (2013). Mild cognitive impairment and dementia in a heterogeneous elderly population: prevalence and risk profile. *African journal of psychiatry*, 16(6). doi: 10.4314/ajpsy.v16i6.58
107. Yang, L., Jin, X., Yan, J., Jin, Y., Xu, S., Xu, Y., et al. (2019). Comparison of prevalence and associated risk factors of cognitive function status among elderly between nursing homes and common communities of China: A STROBE-compliant observational study. *Medicine*, 98(49). doi: 10.1097/MD.0000000000018248
108. Amoo, G., Ogundele, A. T., Olajide, A. O., Ighoroje, M. G., Oluwaranti, A. O., Onunka, G. C., et al. (2020). Prevalence and pattern of psychiatric morbidity among community-dwelling elderly populations in Abeokuta, Nigeria. *Journal of geriatric psychiatry and neurology*, 33(6), 353-362. doi: 10.1177/0891988719892327
109. Bae, S., Shimada, H., Lee, S., Makizako, H., Lee, S., Harada, K., et al. (2017). The relationships between components of metabolic syndrome and mild cognitive impairment subtypes: a cross-sectional study of Japanese older adults. *Journal of Alzheimer's Disease*, 60(3), 913-921. doi: 10.3233/JAD-161230
110. Fernández-Blázquez, M. A., Noriega-Ruiz, B., Ávila-Villanueva, M., Valentí-Soler, M., Frades-Payo, B., Del Ser, T., and Gómez-Ramírez, J. (2021). Impact of individual and neighborhood dimensions of socioeconomic status on the prevalence of mild cognitive impairment over seven-year follow-up. *Aging & Mental Health*, 25(5), 814-823. doi: 10.1080/13607863.2020.1725803
111. Ganguli, M., Chang, C. C. H., Snitz, B. E., Saxton, J. A., Vanderbilt, J., and Lee, C. W. (2010). Prevalence of mild cognitive impairment by multiple classifications: The Monongahela-Youghiogheny Healthy Aging Team (MYHAT) project. *The American Journal of Geriatric Psychiatry*, 18(8), 674-683. doi: 10.1097/JGP.0b013e3181cdee4f
112. González, H. M., Tarraf, W., Schneiderman, N., Fornage, M., Vásquez, P. M., Zeng, D., et al. (2019). Prevalence and correlates of mild cognitive impairment among diverse Hispanics/Latinos: Study of Latinos-Investigation of Neurocognitive Aging results. *Alzheimer's & dementia*, 15(12), 1507-1515. doi: 10.1016/j.jalz.2019.08.202
113. Guaita, A., Vaccaro, R., Davin, A., Colombo, M., Vitali, S. F., Polito, L., et al. (2015). Influence of socio-demographic features and apolipoprotein E epsilon 4 expression on the prevalence of dementia and cognitive impairment in a population of 70–74-year olds: The InveCe. Ab study. *Archives of Gerontology and Geriatrics*, 60(2), 334-343. doi: 10.1016/j.archger.2014.11.006
114. Heywood, R., Gao, Q., Nyunt, M. S. Z., Feng, L., Chong, M. S., Lim, W. S., et al. (2017). Hearing loss and risk of mild cognitive impairment and dementia: findings from the Singapore longitudinal ageing study. *Dementia and geriatric cognitive disorders*, 43(5-6), 259-268. doi: 10.1159/000464281
115. Kivipelto, M., Helkala, E. L., Hänninen, T., Laakso, M. P., Hallikainen, M., Alhainen, K., et al. (2001). Midlife vascular risk factors and late-life mild cognitive impairment: a population-based study. *Neurology*, 56(12), 1683-1689. doi: 10.1212/WNL.56.12.1683
116. Lara, E., Koyanagi, A., Olaya, B., Lobo, A., Miret, M., Tyrovolas, S., et al. (2016). Mild cognitive impairment in a Spanish representative sample: prevalence and associated factors. *International journal of geriatric psychiatry*, 31(8), 858-867. doi: 10.1002/gps.4398
117. Chong, C. P., Shahar, S., Haron, H., and Din, N. C. (2019). Habitual sugar intake and cognitive impairment among multi-ethnic Malaysian older adults. *Clinical interventions in aging*, 1331-1342.
118. Das, S. K., Bose, P., Biswas, A., Dutt, A., Banerjee, T. K., Hazra, A., et al. (2007). An epidemiologic study of mild cognitive impairment in Kolkata, India. *Neurology*, 68(23), 2019-2026. doi: 10.1212/01.wnl.0000264424.76759.e6
119. Juarez-Cedillo, T., Sanchez-Arenas, R., Sanchez-Garcia, S., Garcia-Pena, C., Hsiung, G. Y. R., Sepehry, A. A., et al. (2012). Prevalence of mild cognitive impairment and its subtypes in the Mexican population. *Dementia And Geriatric Cognitive Disorders*, 34(5-6), 271-281. doi: 10.1159/000345251
120. Ding, D., Zhao, Q., Guo, Q., Meng, H., Wang, B., Luo, J., et al. (2015). Prevalence of mild cognitive impairment in an urban community in China: a cross-sectional analysis of the Shanghai Aging Study. *Alzheimer's & Dementia*, 11(3), 300-309. doi: 10.1016/j.jalz.2013.11.002
121. Jia, J., Zhou, A., Wei, C., Jia, X., Wang, F., Li, F., et al. (2014). The prevalence of mild cognitive impairment and its etiological subtypes in elderly Chinese. *Alzheimer's & Dementia*, 10(4), 439-447. doi: 10.1016/j.jalz.2013.09.008
122. Jia, L., Du, Y., Chu, L., Zhang, Z., Li, F., Lyu, D., et al. (2020). Prevalence, risk factors, and management of dementia and mild cognitive impairment in adults aged 60 years or older in China: a cross-sectional study. *The Lancet Public Health*, 5(12), e661-e671. doi: 10.1016/S2468-2667(20)30185-7
123. Anstey, K. J., Cherbuin, N., Eramudugolla, R., Sargent-Cox, K., Easteal, S., Kumar, R., and Sachdev, P. (2013). Characterizing mild cognitive disorders in the young-old over 8 years: prevalence, estimated incidence, stability of diagnosis, and impact on IADLs. *Alzheimer's & Dementia*, 9(6), 640-648. doi: 10.1016/j.jalz.2012.11.013
124. Dimitrov, I., Tzourio, C., Milanov, I., Deleva, N., and Traykov, L. (2012). Prevalence of dementia and mild cognitive impairment in a Bulgarian urban population. *American Journal of Alzheimer's Disease & Other Dementias®*, 27(2), 131-135. doi: 10.1177/1533317512442371
125. Gavrila, D., Antunez, C., Tormo, M. J., Carles, R., Garcia Santos, J. M., Parrilla, G., et al. (2009). Prevalence of dementia and cognitive impairment in Southeastern Spain: the Ariadna study. *Acta Neurologica Scandinavica*, 120(5), 300-307. doi: 10.1111/j.1600-0404.2009.01283.x
126. Han, J. W., So, Y., Kim, T. H., Lee, D. Y., Ryu, S. H., Kim, S. Y., et al. (2017). Prevalence rates of dementia and mild cognitive impairment are affected by the diagnostic parameter changes for neurocognitive disorders in the DSM-5 in a Korean population. *Dementia and Geriatric Cognitive Disorders*, 43(3-4), 193-203. doi: 10.1159/000458408
127. Hänninen, T., Hallikainen, M., Tuomainen, S., Vanhanen, M., and Soininen, H. (2002). Prevalence of mild cognitive impairment: a population‐based study in elderly subjects. *Acta Neurologica Scandinavica*, 106(3), 148-154. doi: 10.1034/j.1600-0404.2002.01225.x
128. Juncos-Rabadán, O., Pereiro, A. X., Facal, D., Rodriguez, N., Lojo, C., Caamaño, J. A., et al. (2012). Prevalence and correlates of cognitive impairment in adults with subjective memory complaints in primary care centres. Dementia and Geriatric Cognitive Disorders, 33(4), 226-232. doi: 10.1159/000338607
129. Kim, K. W., Park, J. H., Kim, M. H., Kim, M. D., Kim, B. J., Kim, S. K., et al. (2011). A nationwide survey on the prevalence of dementia and mild cognitive impairment in South Korea. *Journal of Alzheimer's Disease*, 23(2), 281-291. doi: 10.3233/JAD-2010-101221
130. Limongi, F., Siviero, P., Noale, M., Gesmundo, A., Crepaldi, G., Maggi, S., and Dementia Registry Study Group. (2017). Prevalence and conversion to dementia of Mild Cognitive Impairment in an elderly Italian population. *Aging Clinical and Experimental Research*, 29, 361-370. doi: 10.1007/s40520-017-0748-1
131. Liu, L. Y., Lu, Y., Shen, L., Li, C. B., Yu, J. T., Yuan, C. R., et al. (2022). Prevalence, risk and protective factors for mild cognitive impairment in a population-based study of Singaporean elderly. *Journal of Psychiatric Research*, 145, 111-117. doi: 10.1016/j.jpsychires.2021.11.041
132. Lopez‐Anton, R., Santabarbara, J., De‐la‐Cámara, C., Gracia‐García, P., Lobo, E., Marcos, G., et al. (2015). Mild cognitive impairment diagnosed with the new DSM‐5 criteria: prevalence and associations with non‐cognitive psychopathology. *Acta Psychiatrica Scandinavica*, 131(1), 29-39. doi: 10.1111/acps.12297
133. Luck, T., Riedel-Heller, S. G., Kaduszkiewicz, H., Bickel, H., Jessen, F., Pentzek, M., et al. (2007). Mild cognitive impairment in general practice: age-specific prevalence and correlate results from the German study on ageing, cognition and dementia in primary care patients (AgeCoDe). *Dementia and geriatric cognitive disorders*, 24(4), 307-316. doi: 10.1159/000108099
134. Mohan, D., Iype, T., Varghese, S., Usha, A., and Mohan, M. (2019). A cross-sectional study to assess prevalence and factors associated with mild cognitive impairment among older adults in an urban area of Kerala, South India. *BMJ open*, 9(3), e025473. doi: 10.1136/bmjopen-2018-025473
135. Mooi, C. S., and Hamid, T. A. (2016). Prevalence and factors associated with mild cognitive impairment on screening in older Malaysians. *Dusunen Adam The Journal of Psychiatry and Neurological Sciences*, 29(4), 298. doi: 10.5350/DAJPN2016290401
136. Moretti, F., De Ronchi, D., Palmer, K., Forlani, C., Morini, V., Ferrari, B., et al. (2013). Prevalence and characteristics of mild cognitive impairment in the general population. Data from an Italian population-based study: The Faenza Project. *Aging & Mental Health*, 17(3), 267-275. doi: 10.1080/13607863.2012.732034
137. Noguchi-Shinohara, M., Yuki, S., Dohmoto, C., Ikeda, Y., Samuraki, M., Iwasa, K., et al. (2013). Differences in the prevalence of dementia and mild cognitive impairment and cognitive functions between early and delayed responders in a community-based study of the elderly. *Journal of Alzheimer's Disease*, 37(4), 691-698. doi: 10.3233/JAD-130398
138. Peltz, C. B., Corrada, M. M., Berlau, D. J., and Kawas, C. H. (2012). Cognitive impairment in nondemented oldest-old: prevalence and relationship to cardiovascular risk factors. *Alzheimer's & dementia*, 8(2), 87-94. doi: 10.1016/j.jalz.2011.02.008
139. Robertson, K., Larson, E. B., Crane, P. K., Cholerton, B., Craft, S., McCormick, W. C., et al. (2019). Using varying diagnostic criteria to examine mild cognitive impairment prevalence and predict dementia incidence in a community-based sample. *Journal of Alzheimer's Disease*, 68(4), 1439-1451. doi: 10.3233/JAD-180746
140. Sasaki, M., Kodama, C., Hidaka, S., Yamashita, F., Kinoshita, T., Nemoto, K., et al. (2009). Prevalence of four subtypes of mild cognitive impairment and APOE in a Japanese community. *International Journal of Geriatric Psychiatry: A journal of the psychiatry of late life and allied sciences*, 24(10), 1119-1126. doi: 10.1002/gps.2234
141. Shahnawaz, Z., Reppermund, S., Brodaty, H., Crawford, J. D., Draper, B., Trollor, J. N., & Sachdev, P. S. (2013). Prevalence and characteristics of depression in mild cognitive impairment: the Sydney Memory and Ageing Study. Acta psychiatrica Scandinavica, 127(5), 394–402. <https://doi.org/10.1111/acps.12008>
142. Teh, W. L., Abdin, E., Vaingankar, J. A., Shafie, S., Jeyagurunathan, A., Yunjue, Z., and Subramaniam, M. (2021). Prevalence, Lifestyle Correlates, and Psychosocial Functioning Among Multi-Ethnic Older Adults with Mild Cognitive Impairment in Singapore: Preliminary Findings from a 10/66 Population Study. *The Yale journal of biology and medicine*, 94(1), 73-83.
143. Tsoy, R. T., Turuspekova, S. T., Klipitskaya, N. K., Mereke, A., and Cumming, R. G. (2019). Prevalence of mild cognitive impairment among older people in Kazakhstan and potential risk factors. *Alzheimer Disease & Associated Disorders*, 33(2), 136-141. doi: 10.1097/WAD.0000000000000298
144. Vlachos, G. S., Kosmidis, M. H., Yannakoulia, M., Dardiotis, E., Hadjigeorgiou, G., Sakka, P., et al. (2020). Prevalence of mild cognitive impairment in the elderly population in Greece: results from the HELIAD study. *Alzheimer Disease & Associated Disorders*, 34(2), 156-162. doi: 10.1097/WAD.0000000000000361
145. Liu, C. C., Liu, C. H., Sun, Y., Lee, H. J., Tang, L. Y., and Chiu, M. J. (2022). Rural-urban disparities in the prevalence of mild cognitive impairment and dementia in Taiwan: a door-to-door nationwide study. *Journal of epidemiology*, 32(11), 502-509. doi: 10.2188/jea.JE20200602
146. Su, X., Shang, L., Xu, Q., Li, N., Chen, J., Zhang, L., et al. (2014). Prevalence and predictors of mild cognitive impairment in Xi’an: a community-based study among the elders. *PloS one*, 9(1), e83217. doi: 10.1371/journal.pone.0083217
147. Mías, C. D., Sassi, M., Masih, M. E., Querejeta, A., and Krawchik, R. (2007). Deterioro cognitivo leve: estudio de prevalencia y factores sociodemográficos en la ciudad de Córdoba, Argentina. *Rev Neurol*, 44(12), 733-8.
148. Pedraza, O. L., Montes, A. M. S., Sierra, F. A., Montalvo, M. C., Muñoz, Y., Díaz, J. M., et al. (2017). Mild cognitive impairment (MCI) and dementia in a sample of adults in the city of Bogotá. *Dementia & Neuropsychologia*, 11, 262-269. doi: 10.1590/1980-57642016dn11-030008
149. Sánchez, S. S., Abanto, J., Sanchez-Boluarte, A., Boluarte-Carbajal, A., Sanchez-Coronel, D., Custodio-Capuñay, N., and Samalvides-Cuba, F. (2019). Frequency and associated factors of amnestic mild cognitive impairment at four senior citizen clubs in Lima, Peru. *Dementia & Neuropsychologia*, 13, 321-328. doi: 10.1590/1980-57642018dn13-030009
150. Monteagudo Torres, M., Gómez Viera, N., Martín Labrador, M., Jiménez Fontao, L., Mc Cook, E., and Ruiz García, D. (2009). Evaluación del estado cognitivo de los adultos mayores de 60 años, en un área de salud del Policlínico Docente Plaza de la Revolución. *Revista Cubana de medicina*, 48(3), 59-70.
151. Wesseling, C., Roman, N., Quiros, I., Paez, L., García, V., María Mora, A., et al. (2013). Parkinson's and Alzheimer's diseases in Costa Rica: a feasibility study toward a national screening program. *Global Health Action*, 6(1), 23061. doi: 10.3402/gha.v6i0.23061
152. Li, W., Sun, L., and Xiao, S. (2020). Prevalence, incidence, influence factors, and cognitive characteristics of amnestic mild cognitive impairment among older adult: a 1-year follow-up study in China. *Frontiers in Psychiatry*, 11, 75. doi: 10.3389/fpsyt.2020.00075
153. Rao, D., Luo, X., Tang, M., Shen, Y., Huang, R., Yu, J., et al. (2018). Prevalence of mild cognitive impairment and its subtypes in community-dwelling residents aged 65 years or older in Guangzhou, China. *Archives of Gerontology and Geriatrics*, 75, 70-75. doi: 10.1016/j.archger.2017.11.003
154. Sun, Y., Lee, H. J., Yang, S. C., Chen, T. F., Lin, K. N., Lin, C. C., et al. (2014). A nationwide survey of mild cognitive impairment and dementia, including very mild dementia, in Taiwan. *PloS one*, 9(6), e100303. doi: 10.1371/journal.pone.0100303
155. Xiao, S., Lewis, M., Mellor, D., McCabe, M., Byrne, L., Wang, T., et al. (2016). The China longitudinal ageing study: overview of the demographic, psychosocial and cognitive data of the Shanghai sample. *Journal of Mental Health*, 25(2), 131-136. doi: 10.3109/09638237.2015.1124385
156. Liu, H., Zeng, Q. Z., Zhuang, X. W., and Chen, Y. M. (2018). Investigation analysis of mild cognitive impairment of the elderly in the community of some District in Shanghai and study of the intervention methods and effects. *J Int Psychiatry*, 45(2), 288-91. doi: 10.13479/j.enki.jip.2018.02.030
157. Wu, Y., Yao, J. J., Chen, Z. H., Yang, Q. P., Zhou, D. X., and Feng, W. (2017). Survey on mild cognitive impairment and analysis of its influencing factors among community elderly in Wuxi City. *Modern Prev Med*, 44(2), 259-63. doi: CNKI:SUN:XDYF.0.2017-02-018
158. Chuang, Y. F., Liu, Y. C., Tseng, H. Y., Lin, P. X., Li, C. Y., Shih, M. H., et al. (2021). Urban-rural differences in the prevalence and correlates of mild cognitive impairment in community-dwelling older adults in Taiwan: The EMCIT study. *Journal of the Formosan Medical Association*, 120(9), 1749-1757. doi: 10.1016/j.jfma.2021.03.005
159. Janelidze, M., Mikeladze, N., Bochorishvili, N., Dzagnidze, A., Kapianidze, M., Mikava, N., et al. (2018). Mild cognitive impairment in republic of Georgia. *Gerontology and Geriatric Medicine*, 4, 2333721418771408. doi: 10.1177/2333721418771408
160. Pilleron, S., Jésus, P., Desport, J. C., Mbelesso, P., Ndamba-Bandzouzi, B., Clément, J. P., et al. (2015). Association between mild cognitive impairment and dementia and undernutrition among elderly people in Central Africa: some results from the EPIDEMCA (Epidemiology of Dementia in Central Africa) programme. *British Journal of Nutrition*, 114(2), 306-315. doi: 10.1017/S0007114515001749
161. Vancampfort, D., Stubbs, B., Lara, E., Vandenbulcke, M., Swinnen, N., and Koyanagi, A. (2017). Mild cognitive impairment and physical activity in the general population: Findings from six low-and middle-income countries. *Experimental gerontology*, 100, 100-105. doi: 10.1016/j.exger.2017.10.028
162. Koyanagi, A., Veronese, N., Stubbs, B., Vancampfort, D., Stickley, A., Oh, H., et al. (2019). Food insecurity is associated with mild cognitive impairment among middle-aged and older adults in South Africa: findings from a nationally representative survey. *Nutrients*, 11(4), 749. doi: 10.3390/nu11040749
163. Li, X., Ma, C., Zhang, J., Liang, Y., Chen, Y., Chen, K., et al. (2013). Prevalence of and potential risk factors for mild cognitive impairment in community‐dwelling residents of Beijing. *Journal of the American Geriatrics Society*, 61(12), 2111-2119. doi: 10.1111/jgs.12552
164. Kang, Y., Liu, C., Zhu, H., Chen, W., and Xie, C. (2016). Epidemiological survey and influencing factors of early cognitive impairment in the elderly in Songzi City, 2015. *Pract Prev Med*, 23(12), 1473-1476. doi: 10.3969/j.issn.1006-3110.2016.12.018
165. Huang, Q., Gao, Y., Li, J., Song, Y., Huang, M., Shen, L., et al. (2021). Cognition function and its influencing factors among people aged 55 and above in 4 provinces of China from 2018 to 2019. *Wei Sheng yan jiu= Journal of Hygiene Research*, 50(1), 21-36. doi: 10.19813/j.cnki.weishengyanjiu.2021.01.005
166. Bai, A., Hu, Y., Xu, W., Liu, J., Sun, J., et al. (2021). Prevalence of mild cognitive impairment and its correlation with sarcopenia in different genders among commu- nity-dwelling very old adults in Beijing. *Chin J Clin Healthc*, 24(02), 175-182. doi: 10.3969/J. issn. 1672-679p. 2021.02.006
167. Lu, H., Meng, H., Bai, J., Meng, S., Shang, B., et al. (2022). Status and influencing factors of mild cognitive impairment among the elderly in Chengde Community. *Journal Of Alzheimer's Disease And Related Diseases*, 5(01), 42-45. doi: 10.3969/j.issn.2096-5516.2022.01.008
168. Shi, H., Zhou, X., Zhang, Y., Ye, J., Zhu, M., Chen, Q., et al. (2019). Investigation and Analysis of Early Cognitive Dysfunction among Military Cadres in Southeast Coastal Areaszz. *Military Medical Journal of southeast China*, 21(06), 664-666. doi: 10.3969/i.issn.1672-271X.2019.06.026
169. Liu, B., Shao, H., Peng, Y., and Ding, H. (2005). Investigation and analysis of mild cognitive impairment among elderly people in Luohu District, Shenzhen. *Journal of guangzhou university of traditional chinese Medicine*, 2005(05), 28-30+38. doi: 10.3760/cma.j.issn.1674-2907.2007.28.012
170. Sun, L., Zhang, D., Huang, M., Xiao, Z., and Chen, X. (2013). Fuzhou from the investigation and analysis of epidemiology of mild cognitive impairment in retired cadres. *Global Traditional Chinese Medicine*, 6(S1), 33-35. doi: 10.3969/j.issn.1674-1749.2013.z1.027
171. Hai, S., Dong, B., Zhou, Y., Lin, X., Chen, X., and Li, L. (2010). Prevalence and related risk factors of mild cognitive impairment in elderly people. *Chin J Geront*, 30(03), 397-398. doi: 10.3969/j.issn.1005-9202.2010.03.051
172. Yuan, L., Ding, Y., Yang, D., and Lu, L. (2017). Investigation on the prevalence and influencing factors of mild cognitive impairment among elderly people in Guiyang area. *Guizhou Medical Journal*, 41(08), 867-869. doi: CNKI:SUN:GZYI.0.2017-08-033
173. Ji, N., and Wang, Y. (2017). Prevalence and influencing factors of mild cognitive impairment among elderly people in Kazakh communities. *Chin J Geront*, 37(16), 4113-4115. doi: 10. 3969/j. issn. 1005-9202. 2017.16.092
174. Wang, Z., Ding, L., Liu, L., Li, T., Ma, W., and Zhang, J. (2013). The current status of mild cognitive impairment and its relationship with sexual hormones in community populations aged 55 and above of Hui and Han ethnic groups. *Chin J Nerve Ment Dis*, 39(7), 427-430.
175. Zhao, C., Gao, L., and Fang, J. (2015). The prevalence and influencing factors of mild cognitive impairment among rural elderly people aged 60 and above in Jilin region. *Chinese Rural Health Service Administration*, 35(11), 1434-1437. doi: CNKI:SUN:ZNWS.0.2015-11-029
176. Li, J. (2013). Survey of mild cognitive impairment in old people in community of Jinan city. *Chinese Nursing Research,* 27(21), 2196-2197. doi: 10.3969/j. issn. 1009-6493. 2013. 21.009
177. Pan, H., Wang, J., Pan, C., Duo, W., and Sheng, A. (2020). Prevalence and influencing factors of mild cognitive impairment among elderly people in rural areas of Jinhua City. *Chin J Geront*, 40(20), 4448-4451. doi: 10. 3969/j. issn. 1005-9202. 2020. 20. 056
178. Yu, B., Xu, R., Wei, W., Zhong, W., He, J., Qi, L., et al. (2012). Study of the prevalence of mild cognitive impairment in oldest old male veterans. *Chin J Health Care Med*, 14(02), 125-127. doi: 10.3969/j.issn. 1674-3245.2012.02.015
179. Yu, B., Wang, Y., Tian, J., Wang, C., Ouyang, L., Qi, L., et al. (2002). Study of the prevalence of mild cognitive impairment in elderly male veterans. *Chinese Journal Of Behavioral Medical Science*, 2002(05), 30-32. doi: 10.3760/cma.j.issn.1674-6554.2002.05.010
180. Cai, Y., Huang, W., Yang, J., Ynag, X., Wang, J., Wei, J. (2010). Incidence of mild cogn itive impaiment and its subtypes among the elderly in communities of guiyang city. *China Public Health*, 26(04), 403-405.
181. Chen, X., Fang, G., Ni, R., and Yang, L. (2009). Investigation on the prevalence and influencing factors of mild cognitive impairment in the elderly. *Journal Of Nurses Training*, 24(12), 1117-1120. doi: 10.16821/j.cnki.hsjx.2009.12.029
182. Zhang, B., Wang, J., Sun, H., Wu, H., Ye, G., Song, S., et al. (2013). Exploration of risk factors for the mild cognitive impairment in retired veteran male. *Pract Geriatr*, 27(05), 417-419+423. doi: 10.3969/j. issn. 1003-9198. 2013.05.022
183. Sun, S., Cui, X., and Wang, R. (2008). A survey of mild cognitive impairment in retired elderly people. *China Medical Herald*, 2008(14), 123-124. doi: 10.3969/j.issn.1673-7210.2008.14.085
184. Yu, L., and Yu, Q. (2004). A study on mild cognitive impairment in elderly retired person. *J clin Psychol Med*, 2004(06), 338-339. doi: 10.3969/j.issn.1005-3220.2004.06.008
185. Zhang, X., Cheng, N., Lu, X., Zhou, G., Ren, S., and Duan, L. (2008). The prevalence of mild cognitive impairment in an elderly cohort. *Chin J Geriatric Brain Vessel Dis*, 10(12), 4. doi: 10.3969/j.issn.1009-0126.2008.12.014
186. Jiang, H., Wang, X. M., Huang, K. Y., Zuo, Y. K., Wu, X. M., Gao, Y. F., et al. (2019). Study on prevalence of and influencing factors of mild cognitive impairment among elderly people in communities of Nanning. *Chinese Journal of Disease Control & Prevention*, 313-317. doi: 10.16462/j.cnki.zhibkz.2019.03.014
187. Hu, R., Zhao, S. G., Wang, D. S., Wen, S. R., Niu, G. M., Wang, Z. G., et al. (2012). A prevalence study on mild cognitive impairment among the elderly populations of Mongolian and Han nationalities in a pastoral area of Inner Mongolia. *Zhonghua liu xing bing xue za zhi= Zhonghua liuxingbingxue zazhi*, 33(4), 364-367. doi: 10.3760/cma.j.issn.0254-6450.2012.04.004
188. Guo, X. Y., Zhao, L. M., Li, X. M., and Yang, Q. (2013). Prevalence of mild cognitive impairment among rural Chinese elderly. *Chin J Mult Organ Dis Elderly*, 12(12), 904-7. doi: 10.3969/j.issn.1673-7210.2013.06.044
189. Li, C., Wang, Y., Tian, F., Liang, Y., Zhang, W. (2015). The current situation and influencing factors of mild cognitive impairment among elderly people in rural areas. *Chinese Journal Of Gerontology*, 35(12), 3404-3407. doi: 10.3969/j.issn.1005-9202.2015.12.103
190. Fan, J., Lu, G., Zhang, L., He, B., Yang, Z., and Zhang, Y. (2014). Survey and analysis on mild cognitive impairment in elderly people in countryside. *Chinese Nursing Research*, 28(11), 1314-1315. doi: 10.3969/j.issn.1009-6493.2014.11.013
191. Lv, Q., Zhang, X., Wang, Y., Bo, Y., Gao, Q., Sun, D., et al. (2016). Situation of mild cognitive impairment among the elderly in Pingdingshan community. *Journal of Zhengzhou university (Medical edition)*, 51(04), 510-513. doi: 10.13705/j.issn.1671-6825.2016.04.019
192. Zhang, Z., Wu, H., Zhou, Z., Liu, X., Pan, C., and Li, J. (2021). Mild cognitive impairment among the disabled and related risk factors. *Journal of tongJi university( Medical science)*, 42(02), 254-260. doi: 10. 12289/j. issn. 1008-0392. 20309
193. Yuan, J., Qu, Z. W., Jiang, Q., Yang, Z. D., and Fu, W. Z. (2013). The epidemiological survey of depression disorder and cognitive disorder of the elderly in Shanghai Pudong community. *J Chin Psychiatry*, 23(2), 86-8. doi: CNKI:SUN:LCJS.0.2013-02-007
194. Fang, H., and Sheng, J. (2015). Investigation and Analysis of Mild Cognitive Dysfunction among Elderly People in Zhoujiaqiao Community, Shanghai. *JcM*, 13(10), 17-19. doi: CNKI:SUN:SQYX.0.2015-10-009
195. Pan, J., Chen, L., Wang, J., Xu, H., and Xie, B. (2021). Study on the status of cognitive function in community elderly adults with chronic diseases. *Chinese Journal Of Nursing*, 56(01), 109-115. doi: 10.3761/j.issn.0254-1769.2021.01.018
196. Tao, X. Q., Liao, X., Li, M. Q., Wang, N. B., Li, L., Yang, C. H., and Wu, L. (2016). Epidemiological study on mild cognitive impairment among the elderly in Nanchang community. *Chin J Gerontol*, 36(13), 3283-6. doi: 10.3969 /j.issn.1005-9202.2016.13.092
197. Li, B. (2021). Current status and influencing factors of mild cognitive impairment among elderly people in the community. *Chronic Pathematology J*, 22(04), 625-626+629. doi: 10.16440/j.cnki.1674-8166.2021.04.047.
198. Xu, M., Li, C., He, Y., Wu, Y., Yang, J., Chen, A., et al. (2001). A Prcliminary study of the epidemiology of successful aging and mild cognitive impairment in community elderly. *Shanghai Archives of Psychiatry*, 013(B12), 15-18.
199. Zhou, Q., Ku, M., Lu, X., and Zhou, J. (2020). Status quo of mild cognitive impairment among the frail elderly the community and analysis of influencing factors. *Chinese General Practice Nursing*, 18(23), 2945-2949. doi: 10.12104/j.issn.1674-4748.2020.23.001
200. Qiu, Y., Guan, Q., and Wang, J. (2018). Cognitive characteristics and influencing factors of mild cognitive impairment in community elderly. *Chinese Journal of Clinical Psychology*, 26(2), 313-317. doi: 10.16128/j.cmki.1005-3611.2018.02.022
201. Xia, Y., Ma, X., Wei, X., Dong, L., Zheng, R., He, J., et al. (2011). Cross-sectional Survey of Cognitive Impairment in Neurology Outpatients. *J Med res*, 40(03), 58-60+82. doi: 10.3969/j.issn.1673-548X.2011.03.018
202. Wang, Y. H., and Chen, X. (2015). Research on the cognition of people exposed to the Chinese famine (1959-1961) in early life. *Journal of Chongqing Medical University*, 40(1), 41-45. doi: 10.13406/八cnki.cyxb.000468
203. Yuanyuana, Z., Junyingb, D., Yaxina, Z., Dongmeia, L., Guoqinga, L., & Yana, L. (2020). A sample research on prevalence and risk factors of amnestic mild cognitive impairment in elderly in Shijiazhuang. *Clinical Focus*, 35(7), 610. doi: 10.3969/j.issn.1004-583X.2020.07.006
204. Gao, L. W., Jiang, L., Gao, Y. S., Nie, H. W., and Xu, Y. (2011). Prevalence of mild cognitive impairment and its risk factors among elderly people in Canglang District of Suzhou City. *Occup Health*, 27(23), 2676-8. doi: CNKI:SUN:ZYJK.0.2011-23-006
205. Xue, Z., Qu, C., Ma, F., Wang, T., and Yin, J. (2010). Cross-sectional investugation on mild cognitive impairment of aged people in Taiyuan mine area. *Chin J Pu Blic Health*, 26(04), 407-408. doi: 10.11847/zgggws2010-26-04-13
206. Zhou, L., and Qu, C. (2010). Prevalence of mild cognitive impairment among the elderly in Taiyuan city. *Chinese Rural Health Service Administration*, 30(02), 118-119. doi: CNKI:SUN:ZNWS.0.2010-02-021
207. Liang, W., Qu, C., and Ma, F. (2008). Study on the mild cognitive impairment of the aged people from communities in Taiyuan. *Chinese Journal of Prevention and Control of Chronic Diseases*, 2008(02), 174-175. doi: 10.3969/j.issn.1004-6194.2008.02.021
208. He, X., and Wang, C. (2013). Exploring the prevalence and related risk factors of mild cognitive impairment in the elderly population. *Contemporary Medicine*, 19(04), 47-48. doi: 10.3969/j.issn.1009-4393.2013.4.028
209. Zhang, H., Meng, Y., Zhang, S., Wang, J., Fang, Y., and Zhang, M. (2014). Analysis of the current situation of mild cognitive impairment in elderly people undergoing health examinations in Tangshan City. *Chinese Journal Of Coalindustry Medicine*, 17(02), 319-321. doi: 10.11723/ntgyyx1007-9564201402056
210. Sun, S., Fu, J., Lu, H., Song, N., Li, H., Tan, L., et al. (2012). Survey on Cognitive Impairment of 505Aged People and Its Influencing Factors in Tianjin. *Practical Preventive Medicine*, 19(12), 1891-1893. doi: 10.3969/1-issn.1006-3110.2012.12.052
211. Sun, Y., Zhao, M., Wang, F., Qiang, J., Lei, P., Kong, X., et al. (2019). A cross-sectional study of mild cognitive impairment among elderly senior intellectuals inTianjin, China. *Journal Of Internation Alneurology And Neuro Surgery*, 46(05), 504-508. doi: 10.16636/j.cnki.jinn.2019.05.007.
212. Zhao, J., Wu, T., Han F., Ma, F. Huang G. (2013). Prevalence and influencing factors of MCI among community elderly in Tianjin city. *Chin J Public Health*, 29(1), 1-4. doi: 10.11847/zgggws2013-29-01-01
213. Zhao, J., Wu, T., Han, F., Ma, F., and Huang, G. (2015). Prevalence and influencing factors of MCI among community elderly in Tianjin city. *Chin J Dis Control Prev*, 19(04), 330-333. doi: 10.16462/j.cnki.zhjbkz.2015.04.003
214. Sun, S., Fu, J., Qiao, X., Hui, J., Li, H., Pang, W., et al. (2013). The prevalence of mild cognitive impairment and risk factors of cognitive function of aged people in elderly care institutions of Tianjin. *Modern Preventive Medicine*, 40(10), 1808-1810+1816. doi: CNKI:SUN:XDYF.0.2013-10-067
215. Song, Y., and Li, D. (2019). Status quo and influencing factors of mild cognitive impairment in retired nurses. *Guangxi Medical Journal*, 41(12), 1485-1490. doi: 10. 11675/j. issn. 0253-4304. 2019. 12.04
216. Wu, Y., Yao, J. J., Chen, Z. H., Yang, Q. P., Zhou, D. X., and Feng, W. (2017). Survey on mild cognitive impairment and analysis of its influencing factors among community elderly in Wuxi City. *Modern Prev Med*, 44(2), 259-63. doi: CNKI:SUN:XDYF.0.2017-02-018
217. Fan, Y. A. N. G., Chao, W. A. N. G., and Zong-fu, M. A. O. (2016). Prevalence and influencing factors of mild cognitive impairment among community elderly in Wuhan city. *Chin J Public Health*, 32(12), 1705-1707. doi: 10.11847/zgggws22016-32-12-25
218. Su, X., Hua, Q., Wang, W., Ni, C., Liu, X., Shi, R., et al. (2016). Correlation research between mild cognitive impairmentand chronic disease in community elderly in Xi an. *Chinese Nursing Research*, 30(03), 323-325. doi: 10. 3969/j. issn. 1009-6493. 2016. 03.020
219. Xiang, J., Liang, L., Geng, D., and Tan, C. (2009). Investigation and influencing factors analysis of cognitive impairment among elderly people in Xuzhou community. *Acta Academiae Medicinae Xu Zhou*, 29(11), 730-732.
220. Xu, X. (2010). General demography Research on Cognitive Impairment of the Elderly. *Health Vocational Education*, 28(04), 127-128. doi: 10.3969/j.issn.1671-1246.2010.04.075
221. Ma, L., Li, W., Yue, L., and Xiao, S. (2019). The gender prevalence and influencing factors of amnesic mild cognitive impairment and Alzheimer’s disease: a cross-sectional study of the elderly population in Shanghai community. *Chinese Journal Of Alzheimer’S Disease And Related Disorders*, 2(04), 531-536. doi: 10.3969/j.issn.2096-5516.2019.04.013
222. An, N., Zhuo, S., and Zhang, H. (2020). Analysis of the current status and influencing factors of mild cognitive impairment among the elderly in Zhangjiakou City. *South China J Prev Med*, 46(04), 401-404. doi: 10.12183/j.scjpm.2020.0401
223. Yang, L., Jin, X., Yan, J., Jin, Y., Xu, S., Xu, Y., et al. (2019). Comparison of prevalence and associated risk factors of cognitive function status among elderly between nursing homes and common communities of China: A STROBE-compliant observational study. *Medicine*, 98(49). doi: 10.3760/cma.j.issn.0254-9026.2020.12.022
224. Liu, Y., Li, W., and Jin, H. (2022) .Investigation of the prevalence of mild cognitive dysfunction in the elderly of CNNC and analysis of related factors. *Chin J Neuroim Munol＆ neuro*, 29(03), 234-237.
225. Wang, T., Cao, C., Deng, J., Lian, J., Yan, K., Wang, Z., et al. (2017). The prevalence of mild cognitive impairment in the elderly in Chongqing and its influencing factors. *Chin J Rehabil Theory Pract*, 23(07), 833-838. doi: 10.3969/j.issn. 1006-9771.2017.07.021
226. Wang, T., Cao, C., Liu, Y., Lian, J., Yan, K., Yang, M., et al. (2017). Current status and influencing factors of mild cognitive impairment in the elderly in Chongqing nursing institutions. *Chin J Rehabil Theory Pract*, 23(03):253-256. doi: 10.3969/j.issn.1006-9771.2017.03.002
227. Liu, M., Zhou, C., Long, Y., Xiao, X., Huang, Y., Huang, X., et al. (2021). Investigation of the current situation of mild cognitive impairment in the elderly in Jinwan District, Zhuhai and analysis of influencing factors. *Journal of nursing（China）*, 28(13), 74-78. doi: 10.16460/j.issn1008-9969.2021.13.074
228. Zhou, D., Li, J., Sha, Y., Chai, W., Shen, Y., Gu, A., et al. (2013). Analysis of prevalence and risk factors for mild cognitive dysfunction in hospitalized elderly. *J Medtheor＆Prac*, 2013, 26(02), 147-149. doi: 10.19381/j.issn.1001-7585.2013.02.004
229. Jia, C., Zhang, S., Zhang, M., Li, G., Ma, Y., Tan, J., et al. (2020). Investigation of the current situation of mild cognitive dysfunction of retired cadres stationed in Tianjin and analysis of influencing factors. *J Prev Med chin Pla*, 38(04), 25-27+30. doi: 10.13704/j.cnki.jyyx.2020.04.007
230. Song, W., Li, H., Wang, S., Wang, X., Wang, M. (2011). Analysis and intervention of senile mental disorders in a dry rest center stationed in Kunming. *Soft Science Of Health*, 25(09), 626-628. doi: 10.3969/j.issn.1003-2800.2011.09.014
231. Xu, W., Sun, L., Yang, J., Zhang, L. (2016). Investigation on the prevalence of mild cognitive dysfunction among retired cadres stationed in Qian. *China academic Journal electronicPuBlishing house*, 26(03), 339-341. doi: 10.3969/j.issn.1004-0188.2016.03.044
232. Ma, M., Jiang, L., Kong, X., Cong, L., Wang, J., Xu, Y., et al. (2017). Study on Assessment, Intervention and Follow - up of Cognitive Impairment in the Elderly in Changchun South - lake Community. *Int J Geriatr*, 38(6), 6. doi: 10.3969/j.issn.1674-7593.2017.06.004
233. Zhang, X., and Zeng, H. (2014). Prevalence and Factors Associated with Mild Cognitive Impairment among the Elderly in Changsha Communities. *Chinese General Practice*, 17(09), 1031-1035. doi: 10.3969i.issn.1007-9572.2014.09.014
